# Effect of Ta addition on the structural, thermodynamic and mechanical properties of CoCrFeNi high entropy alloys†

**DOI:** 10.1039/c9ra03055g

**Published:** 2019-05-28

**Authors:** Zhenyu Du, Jie Zuo, Nanyun Bao, Mingli Yang, Gang Jiang, Li Zhang

**Affiliations:** Institute of Atomic and Molecular Physics, Sichuan University Chengdu 610065 China jiezuo@scu.edu.cn lizhang@scu.edu.cn; College of Computer Science, Sichuan University Chengdu 610065 China; Research Center for Materials Genome Engineering, Sichuan University Chengdu 610065 China

## Abstract

Ta addition has considerable effects on the microstructures and mechanical performances of CoCrFeNi alloy systems. Structure search with the special quasirandom structure method and structure identification with first-principles calculations were carried out to investigate the structural, thermodynamic and mechanical properties of CoCrFeNiTa_*x*_ (*x* = 0.0–1.0) high-entropy alloys in the fcc and bcc lattice frameworks. The predicted lattice parameters of identified structures are in agreement with available experiments. Phase transition between the fcc and bcc lattices was predicted for the lowest-energy structures with increasing Ta content. The predicted temperature dependence of specific heat capacity for the identified structures matches well with the Dulong–Petit, Kepp and Debye Models. Both vibration and configuration entropy contribute to the stabilization of alloy systems, while the latter is about 2–3 times greater than the former. The elastic constants and moduli vary with composition and phase structure. Ta atoms have preference to some atoms like Ni, and form relatively strong bonds with adjacent atoms. The introduction of Ta promotes the electron localization and favors the formation of mixed structures.

## Abstract 2

Ta addition alters the microstructures, thermodynamic and mechanical properties of CoCrFeNi alloys.

## Graphical abstract

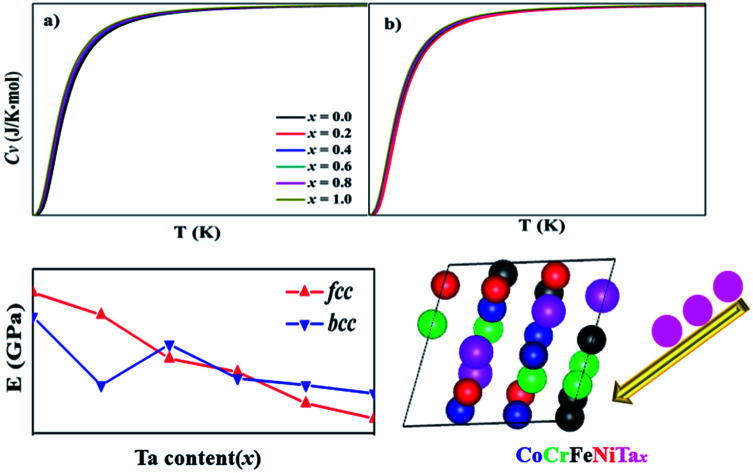

## 1. Introduction

High-entropy alloys (HEAs) have attracted extensive research attention in the past decade, most of which was aimed at developing a series of alloy systems with novel properties.^1–8^ A number of HEAs with unique microstructures,^9,10^ predominant mechanical,^11,12^ great thermal stability,^13^ complex magnetic behavior^14^ and brilliant properties of corrosion resistance^6^ have been reported. Compared with conventional alloys, HEAs have high entropy of mixing that promote the formation of solid solution phase and intermetallic compounds in simple crystal structures. Three kinds structures, face-centered cubic (fcc),^15^ body-centered cubic (bcc)^15^ and hexagonal close-packed (hcp) crystallographic structures,^16^ have so far been characterized for HEAs.

In recent years, HEAs with broad atomic compositions were investigated based on CoCrFeNi systems.^17–21^ Elements like Al, Mo, Ti and Si were successfully added into the CoCrFeNi system to assess their alloying influences on the structures and properties of the multi-component alloys.^22–28^ Wang *et al.*^29^ studied the transition from paramagnetism to superparamagnetism in the amorphous phase for the CoCrFeNiCuTi_*x*_ alloys. Liu *et al.*^30^ concluded that the CoCrFeNiMo_0.3_ HEA exhibits a tensile strength as high as 1.2 GPa and a good ductility of ∼19%. Zhang *et al.*^31^ found that the as-cast structure of Al_*x*_CoCrFeNiTi tends to be a single bcc phase. Ma *et al.*^32^ reported that the compressive yielding strength and Vickers hardness of AlCoCrFeNb_*x*_Ni systems have significant variations with the addition of Nb. On the computational side, Feng *et al.*^33^ investigated the effects of Mn and Al additions on the structural stability and magnetic properties of FeCoNi-based alloys by means of density functional theory (DFT) calculations at Perdew, Burke, and Ernzerhof (PBE) level.^34^ The alloy structures change from fcc to bcc with the increase of Mn and Al content for FeCoNi(MnAl)_*x*_ alloys. First-principles electronic structure calculations at the PBE level were performed by Zaddach *et al.*^35^ to determine the elastic constants and lattice parameters of NiFeCrCoMn alloys, which are in agreement with the mechanical testing and microstructure characterization. Tian *et al.*^36^ predicted that NiCoFeCrAl_*x*_ HEAs have excellent micromechanical properties, and strong metallic and ductility characters when *x* = 1 through *ab initio* calculations using the PBE functional. Those computational studies have proven that computations are an effective approach to identify the microstructures and properties of alloy systems.

Ta, with a high melting point,^37^ was often added to alloys to enhance their structure stability. Zheng *et al.*^38^ investigated the effect of Ta addition on the stress rupture properties and microstructural stability of Ni-based alloys, verifying that the eutectic phase enhances the stress rupture. Stelmakh *et al.*^39^ synthesized the Ta–W solid solution alloy photonic crystals as spectrally selective components for high-temperature energy conversion. Jiang *et al.*^37^ reported the alloying effects of Ta on the microstructures and mechanical properties of CoCrFeNi alloy, finding a high fracture strength of 2.29 GPa for CoCrFeNiTa_0.4_. It has been proven experimentally that Ta addition has great influence on the performances of alloys. However, such influence has been scarcely investigated computationally, especially for the case of Ta-containing HEAs. Computational studies not only predict the microstructures of alloys lack of experimental characterization, but also suggest the correlation between the microstructures and the properties, which are helpful for the design of new alloys with target performances. This work aims to establish the relationship between the microstructures and the performances of CoCrFeNiTa_*x*_ alloys. The microstructures of CoCrFeNiTa_*x*_ alloys, including their interatomic bonding, *g*(*r*), ELF, and formation energy, *etc.*, were computed and correlated with their performances, such as heat capacity, elastic constant and modulus.

## 2. Computational methods

Despite of the single solid solution phase, it is still a great challenge to study HEAs by first-principles calculations because of their multiple-component and complex structures with numerous possible candidates. One strategy to solve this problem is to use the special quasirandom structure (SQS)^40^ method to construct the most disordered HEA structures, which are then sent to subsequent first-principles calculations for further identification. For example, Feng *et al.*^33^ investigated structural stability of quaternary FeCoNiX (X = Al, Mn) alloys using the model structures screened with the SQS method. Wang *et al.*^41^ applied the first-principles phonon method to predict the major phase separations for the refractory VNbMoTaW HEAs which are based on the SQS structures.

In the studied CoCrFeNiTa_*x*_ structures, the fraction of Ta varies between *x* = 0.0 and *x* = 1.0 with an interval of 0.2. It has been noted that lattice structures of HEAs are relevant to their average valence electron concentrations (VEC).^42^ As an indicator of phase structures, VEC is often used to predict that a solid solution adopts fcc or bcc structure.^6^ HEAs usually adopt bcc phase for VEC < 6.87, fcc phase for VEC > 8, and fcc–bcc mixture for 6.87 < VEC < 8. The VEC of CoCrFeNiTa_*x*_ alloys are listed in Table 1, which are about 7.60–8.25, suggesting that the alloys may have both bcc and fcc phases.

**Table 1:** Lattice constants (Å) and averaged valence electron concentration (VEC) of CoCrFeNiTa_*x*_ alloys

*x*	Lattice constants				VEC
	Cal. (bcc)	Exp.	Cal. (fcc)	Exp.	
0.0	3.520	—	3.506	3.575^a^/3.575^b^/3.570^c^	8.25
0.2	3.526	—	3.536	3.586^a^	8.10
0.4	3.585	—	3.586	3.591^a^	7.95
0.6	3.615	—	3.596	—	7.83
0.8	3.619	—	3.623	—	7.71
1.0	3.660	—	3.662	—	7.60

a Ref. 37.

b Ref. 35.

c Ref. 55.

The Alloy-Theoretic Automated Toolkit (ATAT) developed by van de Walle and coworkers^43,44^ was used to generate the SQS structures of the quinary random solid solution CoCrFeNiTa_*x*_ HEAs. Screening the best SQS structure from the numerous candidates of a five-component alloy system is a challenging job. One can hardly figure out the SQS structures for these systems with completely random atomic distribution. In our computations, the screening process was interrupted when the best SQS structure did not change on the list after a long period of time (>100 hours). The lattice vector and atomic positions of the acquired SQS models were given in Table S1 in the ESI.† Ten SQS runs were performed for each composition and all these ten SQS structures were collected for further identification at the first-principles level.

A number of quantities were used to identify the structures of the alloys. Valence electron concentration is computed with1
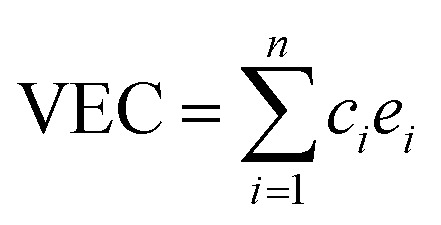
where *c*_*i*_ and *e*_*i*_ are the concentration and number of valence electrons of atom *i* in the cell. To describe the disordered SQS structures, a parameter named total pair distribution function (PDF) is defined as^45–47^2
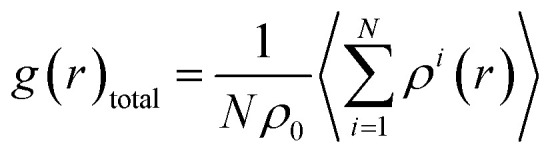
where *N* is the number of atoms in the simulation cell and *ρ*^*i*^(*r*) is the density of atoms in the shell. *ρ*_0_ is the average density of atoms in the SQS models. To compare the atomic arrangements between the two atoms, the partial pair distribution function is also defined as3
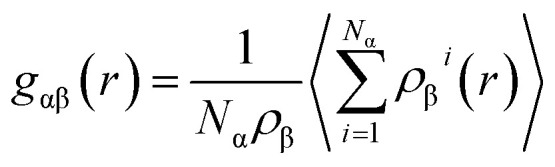
where α and β represent the atomic species.

The structural optimization and energy calculation were carried out for all the SQS screened structures using the DFT calculations with the Vienna Ab initio Simulation Package (VASP).^48^ The projector augmented-wave (PAW) method and standard PBE exchange-correlation functional were employed.^34^ The convergence criteria were set to 10^−8^ eV in energy and 10^−3^ eV Å^−1^ in force in structure optimization. The cut-off energy was selected as 600 eV in the simulations and a Monkhorst–Pack *k*-point mesh of 19 × 19 × 19 was applied for Brillouin zone sampling. It has been shown that these settings^35^ produce reliable results for HEA systems.

The lowest-energy structures screened at the first-principles level for all the studied compositions were further verified with phonon spectroscopy calculations.^49,50^ No imaginary frequencies were found for these structures. The phonon density of states and the heat capacity at constant volume (*C*_v_) and the vibration entropy Δ*S*_vib_ were then computed. The formation energy, Δ*E*_f_, is evaluated with4
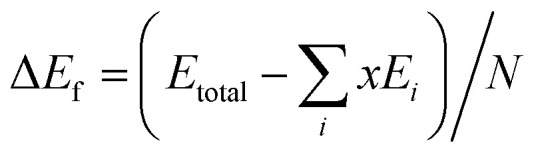
where *E*_total_ is the energy of the SQS structure optimized at the first-principles level, *N* is atom number of SQS supercell and *x* is atom number of element *i* in SQS structure. *E*_*i*_ is the energy of element *i*, which was obtained from the computations on its most stable phase at the same level. The elastic properties of a crystal is calculated using the basic elastic stress–strain relationship:5
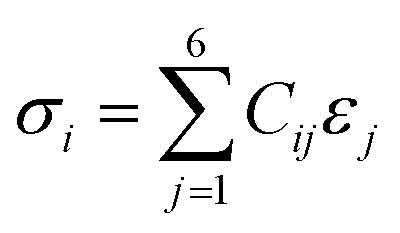
where *σ*_*i*_, *ε*_*j*_, and *C*_*ij*_ are the elastic stress, strain and tensor in the Voigt notation, respectively. *C*_*ij*_ can be derived from this relation by performing six finite distortions of the lattice. Although the atoms are on an fcc (or bcc) lattice, the chemical species distribution in small SQS cells may lead to an anisotropic environment and scattering elastic constants. To overcome this problem, an averaging method^51^ was employed to acquire the *C*_11_, *C*_12_ and *C*_44_ for cubic structures: *C*_11_ = (*c*_11_ + *c*_22_ + *c*_33_)/3, *C*_12_ = (*c*_12_ + *c*_23_ + *c*_13_)/3, and *C*_44_ = (*c*_44_ + *c*_55_ + *c*_66_)/3 in which *c*_*ii*_, *c*_*ij*_ are computed elastic constants. The mechanical properties can be obtained with the elastic constants and the Voigt Reuss (*V*–*R*) average method.^52^ The bulk modulus (*B*) is evaluated with

6 *B* = (*C*_11_ + *C*_12_)/3,

while the shear modulus (*G*) is obtained as a mean of the upper (*G*_V_) and lower (*G*_R_) bounds given by^51,53,54^7
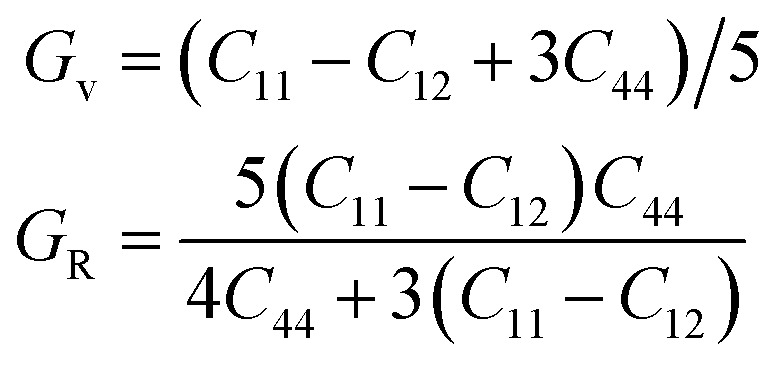


Young's modulus (*E*) can be derived from the *B* and *G*:

8 *E* = (9*BG*)/(3*B* + *G*)

## 3. Results and discussion

The computed lattice parameters of the studied structures are presented in Table 1. The XRD measured parameters are also given for comparison.^35,37,55^ Good agreement between our calculations and measurements were reached. The deviations from the measurements for fcc structures are about 2.0%, 1.4%, and 0.1%, for *x* = 0, 0.2 and 0.4, respectively. Moreover, the lattice parameters increase with Ta content for both bcc and fcc structures. Ta has big atomic size, its addition usually leads to lattice expansion, which has been observed in other experiments.^56–58^

**Fig. 1:**
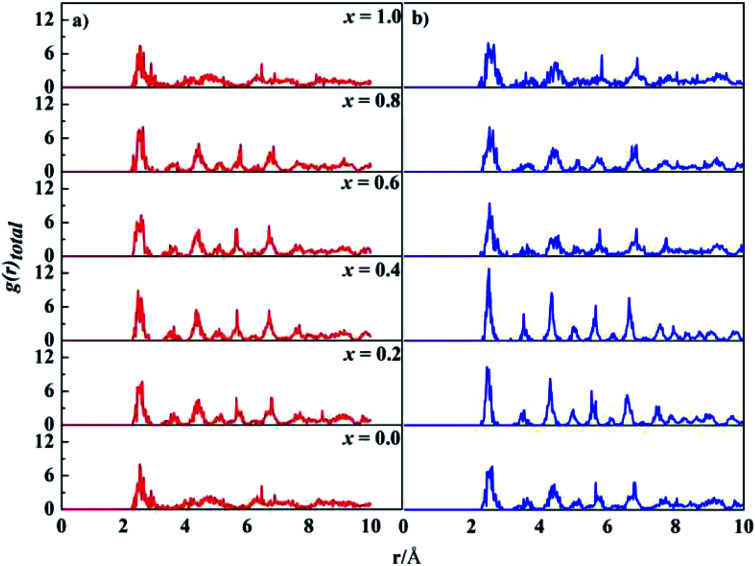
Total pair distribution functions of the DFT-relaxed fcc (left) and bcc (right) structures.

Total PDF, *g*(*r*), measures the number of atoms around given atoms as a function of distance (*r*). Fig. 1 shows the total PDF of the studied CoCrFeNiTa_*x*_ structures. There are several sharp peaks at *r* < 8.0 Å, which implies ordered structures adopted by the atoms at short distances. The *g*(*r*) approaches to 1 when *r* > 8.0 Å, indicating disordered structures in the system at long distance. *g*(*r*) approaches to 1 when *r* increases because the density of atoms in a large concentric layer is close to that of averaged density of atoms of the whole system. The partial PDF, which measures the number of specific atoms around a given atom as a function of *r*. The partial PDFs of all pairs in CoCrFeNiTa_*x*_ are given in Fig. S1 to S12 in the ESI,† which highlight the relative locations of all atoms. Basically, the first peaks of *g*(*r*) occur at about 2.6 Å for all the structures, reflecting the fact that these atoms have similar bondlengths with each other. However, the atomic environments are to some extent different from each other. For example, Co atoms have a tendency to coordinate with each other and with Ni atoms in the fcc structure of *x* = 0.4 (Fig. S5†), while Cr atoms tend to coordinate with Ta and Ni atoms. Moreover, such tendencies are different in the corresponding bcc structures (Fig. S6†) in which Co atoms prefer Ta and Cr atoms, and Fe atoms prefer Cr and Ta atoms. The preferences of interatomic coordinations also change with Ta content. For example, Co atoms prefer Fe, Ta, Co, Ta, Co and Ni atoms, respectively, in the fcc structures when *x* changes from 0 to 1. Ta atoms show some preference in their coordinations. The highest peaks of *x* = 0.2–1.0 are for Ni, Ni, Co, Ni and Cr atoms in the fcc structures, and Ni, Ni, Ni, Fe and Cr atoms in the bcc structures, respectively. These preferences reflect that the diverse microstructures of CoCrFeNiTa_*x*_ alloys.

**Fig. 2:**
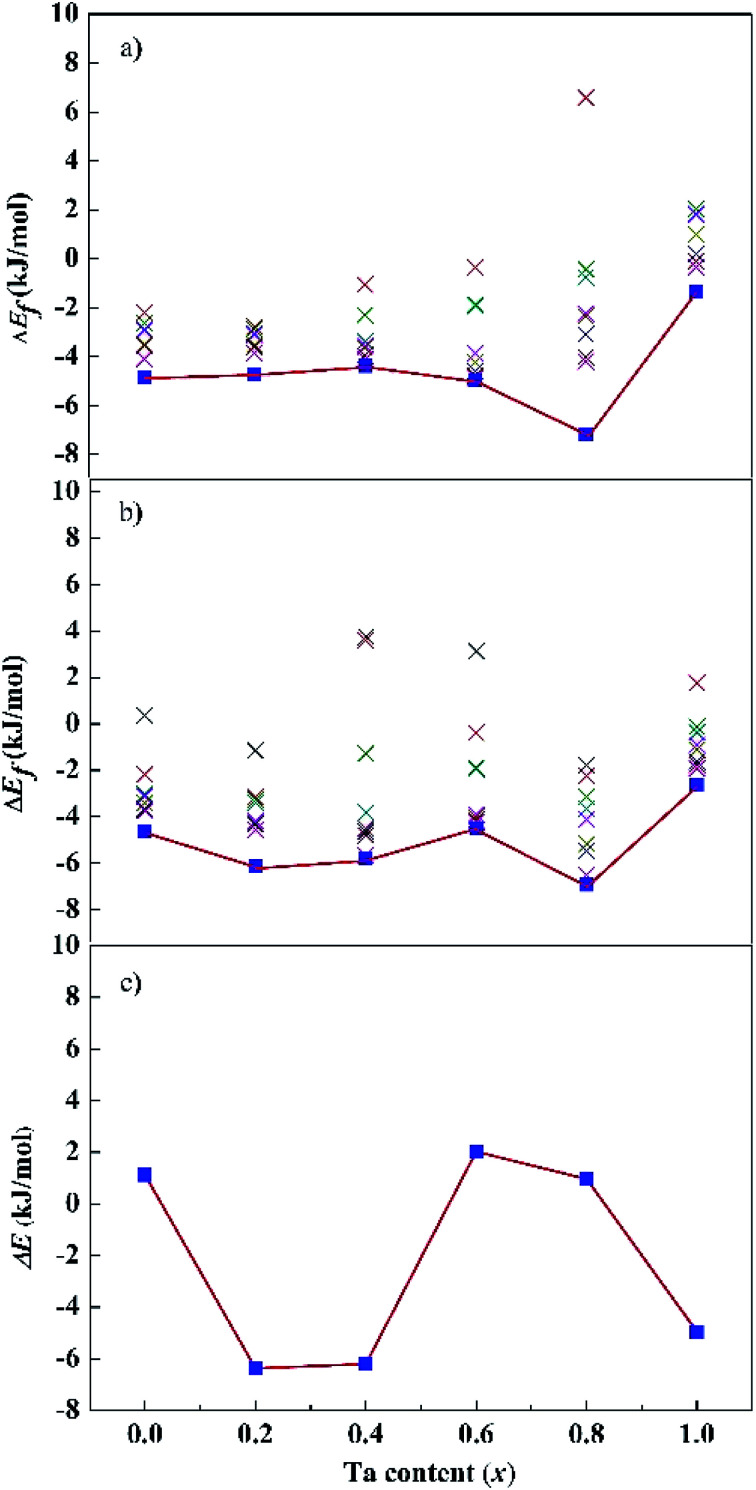
Calculated formation energy of the fcc (a), bcc (b) and energy difference between the bcc and fcc phases (c) of the CoCrFeNiTa_*x*_ alloys. “×” denote the formation energies of other low-lying structures.

Fig. 2a and b show the dependence of formation energies Δ*E*_f_ per atom on Ta content. The Δ*E*_f_ values of all the SQS structures are marked in the figures. Most of them have negative Δ*E*_f_ values except in isolated cases. Our calculations indicate that SQS may predict reasonable structures but repeated runs are necessary to avoid the rare instances. For both the fcc and bcc phases, their Δ*E*_f_ of the lowest-energy structures are negative for all the calculated *x* values. Moreover, the Δ*E*_f_ values vary with Ta content. For the fcc structures, the Δ*E*_f_ values are close for *x* = 0.0–0.6. A drop occurs at *x* = 0.8, followed by an remarkable increase at *x* = 1.0. For the bcc structures, the Δ*E*_f_ values oscillate and reach a bottom at *x* = 0.8, and a peak at *x* = 1.0. The composition of *x* = 0.8 is preferred for both the fcc and bcc structures, while the equimolar alloys have high energy. The relative stability of the alloys with a given composition is measured with their energy difference (Δ*E*) between the bcc and fcc phases, *i.e.*, Δ*E* = (*E*_bcc_ – *E*_fcc_)/*N*, as shown in Fig. 2c. A positive Δ*E* implies a stable fcc structure, and *vice versa*. Δ*E* changes when the Ta content increases from 0.0 to 1.0. The bcc structure is more stable than the corresponding fcc one for *x* = 0.2, 0.4, and 1.0, the fcc structure becomes more stable for *x* = 0.0, 0.6, and 0.8. Our calculations predict that the CoCrFeNiTa_*x*_ alloys may adopt bcc or fcc lattice, or their mixture, depending on the Ta contents. However, one can note that absolute values of Δ*E*_f_ are rather small for all these structures. Jiang *et al.*^37^ found that with increasing Ta content, the microstructure changes from an initial fcc solid solution to a mixed structure in CoCrFeNiTa_*x*_ consisting of bcc and other phases.

One should note that the SQS structures represent the most randomly distributed configuration of atoms in the bcc or fcc lattice at a given composition. These structures were generated within a reasonable period of computing time. Other configurations with similar random atomic distributions may also exist. The characterization of all these possible structures are extremely challenging for multi-component alloys, and we used only one of the most possible structures to explore the thermodynamic and mechanical properties of CoCrFeNiTa_*x*_ alloys.

**Fig. 3:**
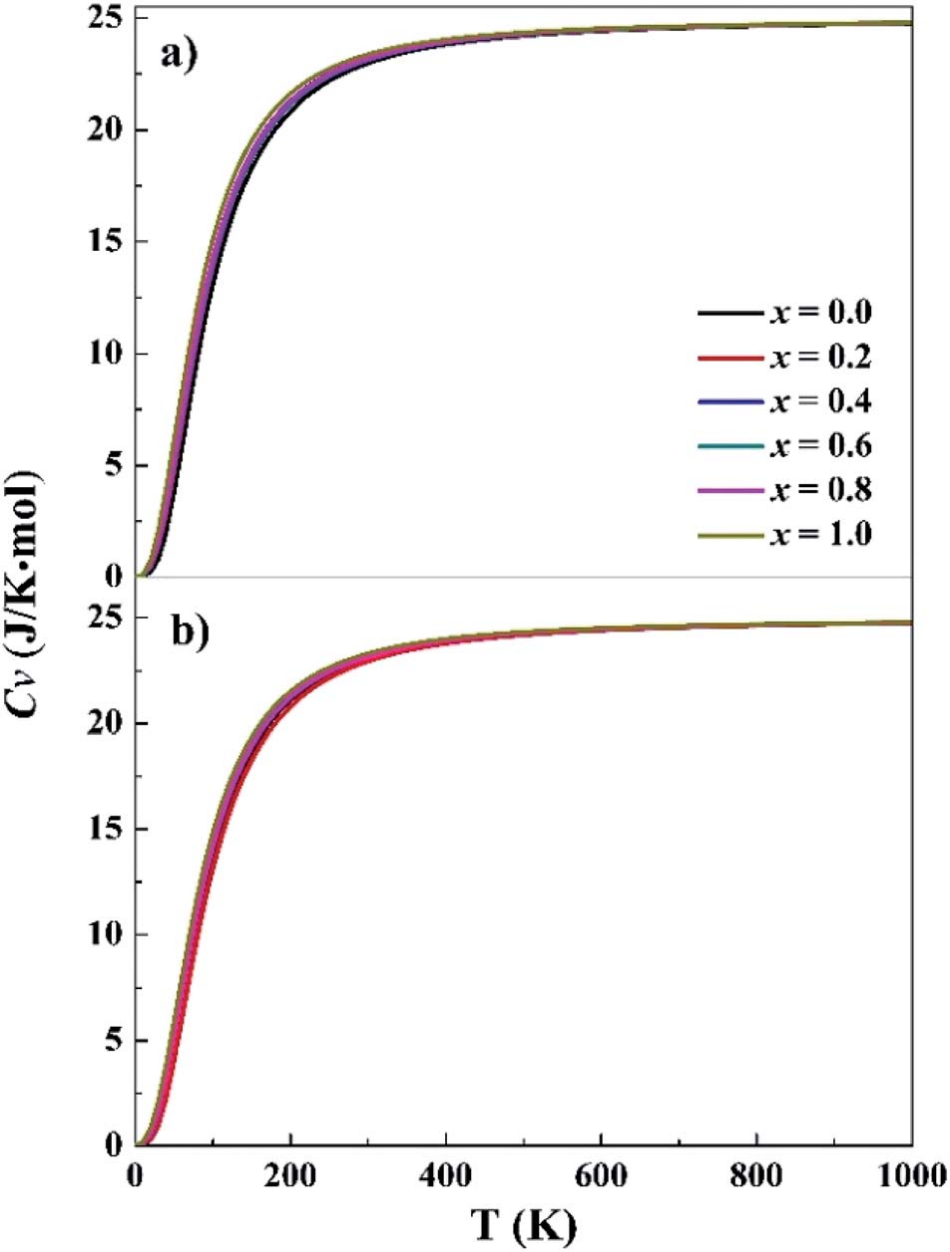
Specific heat capacity at constant volume for the fcc (a) and bcc (b) structures. *x* represents Ta content in the CoCrFeNiTa_*x*_ alloys.

Phonon frequencies were calculated under the harmonic approximation for the lowest-energy bcc and fcc structures. The computed density of phonon states (PHDOS) are given in Fig. S13 in the ESI.† No imaginary frequency was noted in the PHDOS for all the structures, verifying that the identified structures were local minimum on the potential energy surfaces. In addition, the PHDOS were used to calculate heat capacity, *C*_v_, as a function of temperature, as presented in Fig. 3. The *C*_v_ values appear to be quite similar for the studied alloys, increasing rapidly at low temperature (<150 K), increasing slowing at 150–400 K and approaching to 25 J K^−1^ mol^−1^ at high temperature. For *T* near to 0 K, the valence electrons contribute mainly to *C*_v_, making it proportional to *γT*, where *γ* = 10^−4^. Both phonons and electrons make contribute to the total *C*_v_ when *T* < *θ*_D_ (Debye temperature). When *T* ≫ *θ*_D_, *C*_v_ = 3*Nk* ≈ 25 J K^−1^ mol^−1^. The *C*_v_ variations of CoCrFeNiTa_*x*_ are consistent with the prediction of Dulong–Petit, Kepp and Debye Models.^59–61^

**Fig. 4:**
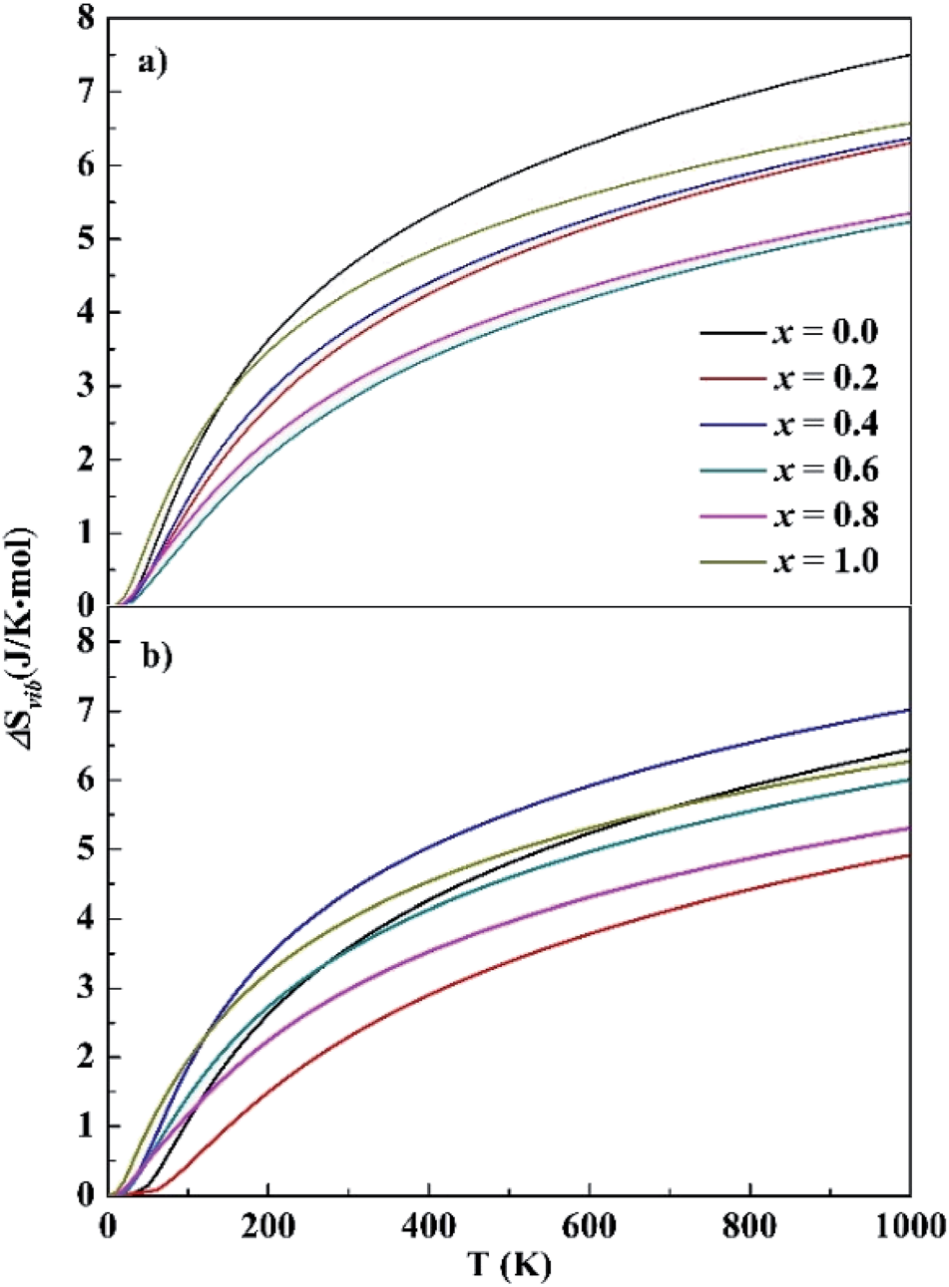
Calculated vibrational entropy of the fcc (top) and bcc (bottom) structures. *x* represents the Ta content in CoCrFeNiTa_*x*_ alloys.

For HEA systems entropy changes are usually dominant because of their rather small formation energies. In contrast to the small Δ*E*_f_ values presented above, relatively large entropy contributions were noted in the CoCrFeNiTa_*x*_ systems. The entropy change contains two parts, vibration (Δ*S*_vib_) and configuration (Δ*S*_con_). The Δ*S*_vib_ values of CoCrFeNiTa_*x*_, which were computed based on the phonon frequencies at the first-principles level, are given in Fig. 4. The Δ*S*_vib_ exhibits similar variations with temperature, close to zero at low temperature and increasing gradually with temperature. The magnitudes of Δ*S*_vib_ vary with Ta content. The differences are small at low temperature, increase with temperature between 0–250 K, but become almost unchanged at *T* > 300 K. The largest differences are about 2.5 J K^−1^ mol^−1^ for the fcc structures between *x* = 0.0 and 0.6, and for the bcc structures between *x* = 0.2 and 0.4. The configurational entropy Δ*S*_con_ and Δ*S*_vib_ at 300 K of the studied alloys are presented in Table 2. Both Δ*S*_con_ and Δ*S*_vib_ values are positive, implying that the formation of the alloys is a process of entropy increment. The magnitudes of Δ*S*_con_ are about 2–3 times greater than those of Δ*S*_vib_, indicating that configurational entropy plays a dominant role in stabilizing the alloys. Although Δ*S*_vib_ becomes greater at high temperature, its contribution to total entropy is still smaller than the Δ*S*_con_. A high entropy change promotes the extent of confusion in alloys and reduces the Gibbs free energy, favoring the random distribution of different elements in crystal lattice.

**Table 2:** Calculated vibration entropy (Δ*S*_vib_) at 300 K and configurational entropy (Δ*S*_con_) of the CoCrFeNiTa_*x*_ alloys

*x*	Δ*S*_vib_ (J K^−1^ mol^−1^)		Δ*S*_con_ (J K^−1^ mol^−1^)
	bcc	fcc	
0.0	3.58	4.62	11.53
0.2	2.29	3.61	12.57
0.4	4.39	3.78	13.01
0.6	3.55	2.81	13.24
0.8	2.98	3.02	13.35
1.0	3.99	4.27	13.38

The computed averaged elastic constants *C*_11_, *C*_12_ and *C*_44_, as well as the Cauchy pressure (*C*_12_–*C*_44_) and the Zener ratio *A*_z_ = 2*C*_44_/(*C*_11_ − *C*_12_) of CoCrFeNiTa_*x*_ are presented in Table 3. The dynamical stability conditions, *i.e.*, *C*_44_ > 0, *C*_11_ > |*C*_12_| and *C*_11_ + 2*C*_12_ > 0,^62^ are satisfied by the presented lowest-energy fcc and bcc structures. For the structures with the same compositions, their elastic constants are different for the fcc and bcc ones. Some components, for example, *C*_11_ of *x* = 0.2 and *C*_12_ of *x* = 0.8, differ remarkably between the two phases. In the same phase, either fcc or bcc, the elastic constants are also different for the structures with different compositions. For example, *C*_44_ of the fcc structures varies between 66 and 160 GPa for *x* = 0–1, while the *C*_44_ of the bcc structures varies between 86 and 148 GPa. Therefore, the elastic constants of CoCrFeNiTa_*x*_ alloys vary with their phase structures and compositions. Positive Cauchy pressure is featured by ductile alloys, while negative Cauchy pressure is a signature of brittle alloys.^63^ The fcc structure of *x* = 0 and the bcc structures of *x* = 0, 0.4 and 0.8 are brittle, the rest of the structures are ductile. *A*_z_ is used to predict the elastic anisotropy of materials. *A*_z_ = 1 represents completely elastic isotropy, and its deviation from 1 measures the degree of elastic anisotropy.^64^ The predicted *A*_z_ values of most structures are far from 1, verifying the anisotropic distribution of atoms in those lattice framework, as noted in the SQS structures presented in Table S1.†

**Table 3:** Computed elastic constants (*C*_11_, *C*_12_ and *C*_44_), the Cauchy pressure (*C*_12_–*C*_44_) and *A*_z_ for the CoCrFeNiTa_*x*_ alloys

*x*	*C*_11_	*C*_12_	*C*_44_	*C*_12_–*C*_44_	*A*_z_
**fcc**					
0.0	321	130	160	−30	1.68
0.2	392	108	105	+3	0.74
0.4	323	121	98	+23	0.97
0.6	287	142	110	+32	1.52
0.8	244	117	97	+20	1.52
1.0	297	126	66	+60	0.77

**bcc**					
0.0	304	134	148	−14	1.75
0.2	317	146	87	+59	1.02
0.4	318	76	103	−27	0.85
0.6	271	120	105	+15	1.39
0.8	275	62	86	−24	0.80
1.0	263	91	88	+3	1.02

**Fig. 5:**
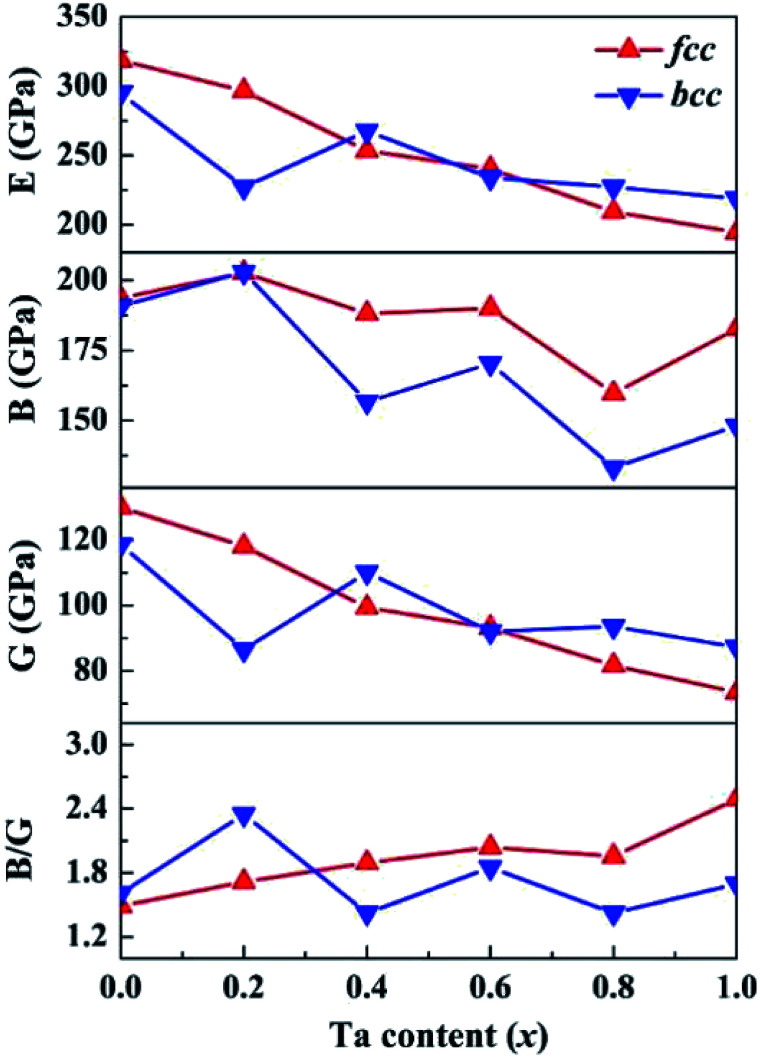
Calculated shear modulus (*G*), Young's modulus (*E*), bulk modulus (*B*), and *B*/*G* of the CoCrFeNiTa_*x*_ alloys.

Based on the computed elastic constants, we further evaluated the shear modulus *G*, Young's modulus *E*, bulk modulus *B*, and Pugh ratio *B*/*G* of the CoCrFeNiTa_*x*_ alloys, which are presented in Fig. 5. Tian *et al.* predicted 207, 280 and 110 GPa for the *B*, *E* and *G* of CoCrFeNi alloys using DFT calculations.^36^ The corresponding values are 194, 319 and 130 GPa, which are in good agreement with Tian's computations. The moduli vary in a narrow ranges, 160–203 GPa for *B*, 73–130 GPa for *G* and 194–319 GPa for *E* of the fcc structures. The corresponding variations are 133–203, 87–119 and 219–295 GPa phase for the bcc structures. The *E*, *G* and *B* values exhibit a decreasing trend with Ta content, but the decreases are small. Moreover, the *E*, *G* and *B* values are dependent on the phase structure and composition. These moduli are to some extent different between the fcc and bcc structures with the same composition. *B*/*G* is also an empirical index to estimate the ductile-brittle behavior of alloys.^65^ Materials with *B*/*G* > 1.75 are ductile, whereas those with lower *B*/*G* values are considered to be brittle. According to the computed results, with the addition of Ta, the fcc structures become more ductile, whereas the bcc structures for *x* = 0.4 and 0.8 are brittle, which complies with the prediction with Cauchy pressure.

**Fig. 6:**
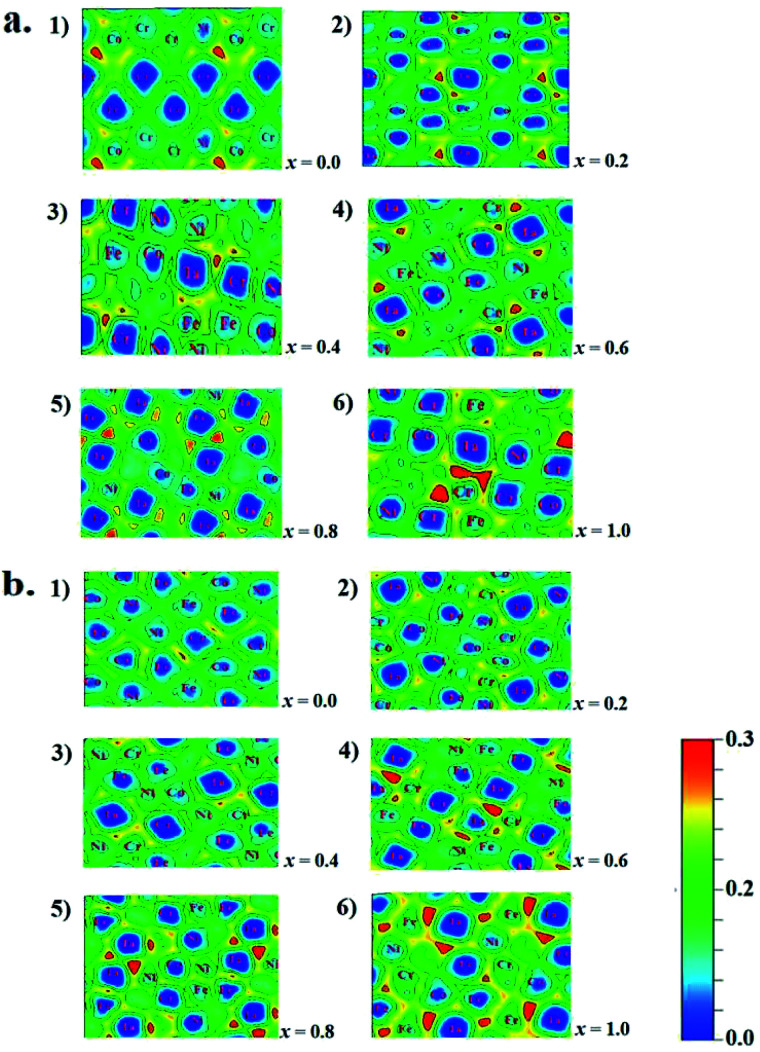
Electron localization function of fcc (a) and bcc (b) of CoCrFeNiTa_*x*_ alloys. (1–6) Denote the ELF values of the structures of *x* = 0.0 to 1.0, respectively.

The microstructures, thermodynamic and mechanical properties of CoCrFeNiTa_*x*_ alloys are related to their electronic structures. Analysis on their electronic structures may shed light on their composition- and structure-dependent variations that are presented above. Electron localization function (ELF) is often used to analyze the interatomic interaction in alloy systems. ELF illustrates electron density among adjacent atoms that is used to classify the interatomic bonds and measure their bonding strengths.^66^ To highlight the effect of Ta addition on interatomic interaction, we calculated the ELF on the Ta-containing facets of the studied structures, as given in Fig. 6. In the figures ELF = 0 and 1 corresponds to a completely delocalized state and a perfect localized state, respectively. The ELF values are about 0.20–0.30 in the regions around Ta atoms, indicating that they has a great tendency to localize electrons. The electron localization tends to be enhanced with increasing Ta content for both the fcc and bcc structures. Two structures of *x* = 1.0 exhibit the largest ELF values around the Ta atoms. Although the enhanced localization strengthens the interatomic interaction between Ta and other atoms, it favors to form other phases. Similar interatomic interaction strengths are one of the features in HEA systems. The strong interaction between Ta and other atoms may disequilibrate the random distribution in alloys. Other experiments^37,67^ have verified that the addition of Ta in CoCrFeNi tend to form a mixed structures.

The band structures of CoCrFeNiTa_*x*_ are presented in Fig. S14 and S15 in the ESI.† All these alloys exhibit metallicity, as illustrated by their dense distribution at the Fermi level. The band structures are similar for all the fcc and bcc structures except Ta-free sample shows some differences in its conductive band. Fig. S16† depicts the total density of states (DOS) of CoCrFeNiTa_*x*_. The overall DOS are similar for the alloys. The spin-up and spin-down distributions are almost symmetric and only some small changes are noted for the alloys with Ta addition.

## 4. Conclusions

Using a combined SQS structure search and first-principles calculation approach, we have investigated the microstructures, thermodynamic and mechanical properties of CoCrFeNiTa_*x*_ (*x* = 0.0–1.0) high entropy alloys. The SQS method was used to construct the disordered candidate structures, which were further verified with first-principles calculations. The identified structures have their lattice constants in good agreement well with the measures. The total and partial PDF reveal that the atoms have different environments from each other, varying with composition and phase structures. Ta atoms have preferences to coordinating with specific atoms. The *C*_v_ variations with temperature are similar for the studied alloys, and are consistent with the prediction of Dulong–Petit, Kepp and Debye Models. Structure transition between the fcc and bcc structures was noted with Ta addition, but the formation energies are small for both phases. The magnitudes of Δ*S*_con_ are about 2–3 times greater than their Δ*S*_vib_ counterparts. It is configurational entropy that plays a crucial role in stabilizing the alloys. The computed elastic constants and moduli revealed that the mechanical properties of the CoCrFeNiTa_*x*_ alloys vary with Ta content and phase structure, but the variations are relatively small. Using the computed moduli, the fcc structure of *x* = 0 and the bcc structures of *x* = 0, 0.4, and 0.8 were predicted to be brittle, and the rest of the structures ductile. The Ta addition alters the electron localization that has considerable influence on the equilibrium among atoms in HEA systems because Ta atoms have relatively stronger interaction with some specific atoms in the systems. Our computations revealed the variations in microstructures, thermodynamic and mechanical properties of the CoCrFeNiTa alloys, which would be helpful for their design and preparation with target performances.

## Conflicts of interest

There are no conflicts to declare.

## Supplementary Material

RA-009-C9RA03055G-s001
