# Supplementary material for: Effect of Ta addition on the structural, thermodynamic and mechanical properties of CoCrFeNi high entropy alloys
Source: RSC Adv. 2019 May 28;9(29):16447–54. doi: 10.1039/c9ra03055g (PMC9119285; doi:10.1039/c9ra03055g)
Supplement: RA-009-C9RA03055G-s001 [file RA-009-C9RA03055G-s001.pdf]

Electronic Supplementary Material (ESI) for RSC Advances.
This journal is © The Royal Society of Chemistry 2019

Supplementary Information

of

Effect of Ta addition on the structural, thermodynamic and mechanical properties of CoCrFeNi high entropy alloys

Zhenyu Du [a], Jie Zuo [b,*], Nanyun Bao [a], Mingli Yang [c], Gang Jiang [a] and Li Zhang [a,*]

[a] Institute of Atomic and Molecular Physics, Sichuan University, Chengdu 610065, China

[b] College of Computer Science, Sichuan University, Chengdu 610065, China

[c] Research Center for Materials Genome Engineering, Sichuan University, Chengdu 610065, China

[*] Corresponding authors: jiezuo@scu.edu.cn (J. Z.) and lizhang@scu.edu.cn (L. Z.)

Table S1 SQSs of CoCrFeNiTa$_x$ (x = 0.0, 0.2, 0.4, 0.6, 0.8 and 1.0) structures containing 20/21/22/23/24/25 atoms, respectively. Lattice vectors and atomic positions are given in Cartesian coordinates in Å. Atomic positions represents the ideal, unrelaxed sites.

	CoCrFeNi		CoCrFeNiTa$_{0.2}$	
	bcc	*fcc*	*bcc*	*fcc*
Lattice vectors	(1.5, 1.5, -0.5)	(1.5, 0.5, 0.0)	(1.5, -0.5, -0.5)	(-2.0, 1.5, -0.5)
	(1.0, -2.0, 0.0)	(-0.5, 1.5, 0.0)	(0.0, -2.0, 1.0)	(1.5, -2.0, -0.5)
	(-1.0, 0.0, -2.0)	(0.0, 0.0, 2.0)	(-0.5, -1.5, -2.5)	(-1.0, -1.0, 1.0)
Atomic positions	(0.0, -1.0, -2.0) Ni	(1.0, 2.0, 1.0) Ni	(0.5, -3.5, -1.5) Cr	(-2.0, 0.0, -0.0) Ni
	(1.0, 1.0, -2.0) Cr	(0.0, 0.5, 1.5) Ni	(0.0, -3.0, -1.0) Co	(-1.5, -0.5, 0.0) Ni
	(0.5, -1.5, -1.5) Co	(1.0, 2.0, 2.0) Co	(0.0, -2.0, -2.0) Ni	(-1.0, -1.0, 0.0) Fe
	(1.5, 0.5, -1.5) Ni	(0.0, 0.5, 0.5) Cr	(1.0, -3.0, -2.0) Fe	(-1.0, -0.5, 0.5) Fe
	(2.0, 0.0, -1.0) Fe	(0.0, 1.0, 1.0) Cr	(0.5, -2.5, -1.5) Co	(-0.5, -1.5, 0.0) Ni
	(0.0, 0.0, -2.0) Fe	(0.0, 1.5, 1.5) Co	(0.0, -2.0, -1.0) Ta	(-0.5, -1.0, 0.5) Ta
	(1.0, -1.0, -2.0) Cr	(0.5, 0.5, 1.0) Fe	(1.0, -3.0, -1.0) Fe	(0.0, -2.0, 0.0) Fe
	(0.5, -0.5, -1.5) Ni	(0.5, 1.0, 1.5) Ni	(0.5, -2.5, -0.5) Cr	(-1.5, 0.5, 0.0) Co
	(0.0, 0.0, -1.0) Co	(1.0, 0.5, 1.5) Fe	(0.0, -2.0, -0.0) Ni	(-1.0, -0.5, -0.5) Ni
	(1.0, -1.0, -1.0) Cr	(0.0, 1.0, 2.0) Cr	(1.0, -2.0, -2.0) Ni	(-1.0, 0.0, 0.0) Cr
	(0.5, -0.5, -0.5) Fe	(0.0, 1.5, 0.5) Fe	(0.5, -1.5, -1.5) Ni	(-0.5, -1.0, -0.5) Cr
	(1.5, -0.5, -2.5) Cr	(0.5, 0.5, 2.0) Cr	(0.0, -1.0, -1.0) Co	(-0.5, -0.5, 0.0) Co
	(1.0, 0.0, -2.0) Ni	(0.5, 1.0, 0.5) Co	(1.0, -2.0, -1.0) Ni	(0.0, -1.0, 0.0) Fe
	(0.5, 0.5, -1.5) Fe	(0.5, 1.5, 1.0) Fe	(0.5, -1.5, -0.5) Cr	(0.5, -1.5, 0.0) Co
	(1.5, -0.5, -1.5) Ni	(1.0, 0.5, 0.5) Co	(0.0, -1.0, -0.0) Cr	(-2.5, 0.0, 0.5) Cr

	(1.0, 0.0, -1.0) Co	(1.0, 1.0, 1.0) Cr	(1.0, -2.0, -0.0) Fe	(-2.0, -0.5, 0.5) Cr
	(0.5, 0.5, -0.5) Co	(1.0, 1.5, 1.5) Ni	(0.0, -3.0, -2.0) Fe	(-1.5, -1.0, 0.5) Co
	(1.5, -0.5, -0.5) Fe	(0.5, 1.5, 2.0) Co	(1.0, -1.0, -1.0) Co	(-1.0, -1.5, 0.5) Cr
	(1.0, 1.0, -1.0) Cr	(1.0, 1.0, 2.0) Ni	(0.5, -0.5, -0.5) Co	(-1.5, -1.5, 0.0) Ni
	(1.5, 0.5, -0.5) Co	(1.0, 1.5, 0.5) Fe	(1.0, -4.0, -2.0) Fe	(-0.5, -2.0, 0.5) Fe
			(0.5, -2.5, -2.5) Cr	(0.0, -2.5, 0.5) Co

	$CoCrFeNiTa_{0.4}$		$CoCrFeNiTa_{0.6}$	
	bcc	*fcc*	*bcc*	*fcc*
Lattice vectors	(-1.5, -1.5, 0.5)	(0.5, 0.0, -1.5)	(-1.5, 0.5, -1.5)	(-0.5, 1.5, 0.0)
	(1.5, -0.5 1.5)	(-1.0, 1.0, -1.0)	(0.0, -2.0, -1.0)	(0.0, 0.5, -1.5)
	(-1.0, 2.0, 1.0)	(1.0, 2.0, 0.0)	(-2.0, -1.0,1.0)	(-2.5, 0.0, -0.5)
Atomic positions	(-1.0, -1.0, 1.0) Ni	(0.5, 3.0, -2.5) Cr	(-3.0, -2.0, -1.0) Fe	(-2.5, 0.5, -1.0) Co
	(-1.5, -0.5,1.5) Co	(0.0, 0.5, -0.5) Co	(-2.5, -2.5, -0.5) Co	(-2.5, 1.0, -0.5) Co
	(-0.5, -1.5, 1.5) Ni	(-0.5, 1.0, -1.5) Cr	(-2.5, -1.5, -1.5) Ni	(-2.0, 0.5, -0.5) Fe
	(-0.5, -0.5, 0.5) Cr	(-0.5, 1.5, -1.0) Co	(-3.0, -1.0, -1.0) Ta	(-2.5, 1.0, -1.5) Fe
	(-1.0, 0.0, 1.0) Cr	(0.0, 0.5, -1.5) Cr	(-2.0, -2.0, -1.0) Ni	(-2.5, 1.5, -1.0) Co
	(-1.5, 0.5, 1.5) Cr	(0.0, 1.0, -1.0) Ni	(-2.5, -1.5, -0.5) Ni	(-2.0, 0.5, -1.5) Ni
	(-1.0, 1.0, 2.0) Fe	(0.5, 1.5, -2.0) Cr	(-1.5,-2.5, -0.5) Co	(-2.0, 1.0, -1.0) Fe
	(-0.5, -0.5, 1.5) Fe	(0.5, 0.5, -1.0) Co	(-2.0, -2.0, -0.0) Fe	(-2.0, 1.5, -0.5) Cr
	(-1.0, 0.0, 2.0) Fe	(0.5, 1.0, -0.5) Ni	(-1.5, -1.5, -1.5) Ta	(-1.5, 0.5, -1.0) Ta
	(0.0, -1.0, 2.0) Ta	(0.0, 1.0,-2.0) Fe	(-2.0, -1.0, -1.0) Co	(-1.5, 1.0, -0.5) Ni
	(-0.5, -0.5, 2.5) Ni	(0.0, 1.5, -1.5) Ni	(-2.5, -0.5, -0.5) Cr	(-1.0, 0.5, -0.5) Ta

	(-1.0, 0.0, 3.0) Cr	(0.0, 2.0, -1.0) Fe	(-1.0, -2.0, -1.0) Ni	(-3.0, 2.0, -2.0) Cr
	(-0.5, 0.5, 0.5) Ni	(0.5, 1.0, -1.5) Ta	(-1.5,-1.5 -0.5) Fe	(-2.0, 1.5, -1.5) Fe
	(-1.0, 1.0, 1.0) Co	(0.5, 1.5, -1.0) Fe	(-2.0, -1.0, -0.0) Co	(-1.5, 1.0, -1.5) Fe
	(0.0, 0.0, 1.0) Ni	(0.5, 2.0, -0.5) Cr	(-1.5, -0.5, -1.5) Fe	(-1.5, 1.5, -1.0) Cr
	(-0.5, 0.5, 1.5) Co	(0.0, 2.0, -2.0) Fe	(-2.0, 0.0, -1.0) Cr	(-1.0, 0.5, -1.5) Ni
	(0.5, -0.5, 1.5) Fe	(0.0, 2.5, -1.5) Fe	(-1.0, -1.0, -1.0) Cr	(-1.0, 1.0, -1.0) Cr
	(0.0, 0.0, 2.0) Fe	(0.5, 2.0, -1.5) Co	(-1.5, -0.5, -0.5) Co	(-1.0, 1.5, -0.5) Ni
	(-0.5, 0.5, 2.5) Co	(0.5, 2.5, -1.0) Ta	(-0.5, -1.5, -0.5) Cr	(-0.5, 0.5, -1.0) Ni
	(-0.5, 1.5, 1.5) Ta	(1.0, 1.5, -1.5) Ni	(-1.0, -1.0, -0.0) Fe	(-0.5, 1.0, -0.5) Co
	(0.5, 0.5, 1.5) Cr	(1.0, 2.0, -1.0) Co	(-1.0, 0.0, -1.0) Ta	(-1.0, 1.5, -1.5) Co
	(0.0, 1.0, 2.0) Co	(0.5, 2.5, -2) Ni	(-0.5, -0.5, -0.5) Ni	(-0.5, 1.0, -1.5) Cr
			(-3.5, -2.5, -1.5) Cr	(-0.5, 1.5, -1.0) Ta

	$CoCrFeNiTa_{0.8}$		$CoCrFeNiTa_{1.0}$	
	bcc	*fcc*	*bcc*	*fcc*
Lattice vectors	(-1.0, -1.0, -2.0)	(1.5, 0.0, 0.5)	(-1.0, 2.0, 1.0)	(1.0, -1.0, -1.0)
	(-1.0, 2.0, 1.0)	(-0.5, -1.5, 0.0)	(-2.0, -1.0 ,1.0)	(1.5, -0.5, 1.0)
	(2.0, 1.0, -1.0)	(0.5, 0.0, -2.5)	(1.5, -0.5, 1.5)	(-0.5, -2.0, 0.5)
Atomic positions	(-0.5, -0.5, -1.5) Co	(0.0, -1.0, 0.0) Fe	(-1.5, -0.5, 1.5) Cr	(1.5, -2.5, 1.0) Ta
	(1.0, 1.0, -2.0) Ta	(0.0, -1.0, -1.0) Ni	(-2.0, 0.0, 2.0) Ta	(0.0, -1.5, 0.5) Co
	(0.0, 0.0, -2.0) Co	(0.0, -0.5, -0.5) Fe	(-1.0, -1.0, 2.0) Ni	(0.5, -2.5, 0.0) Cr
	(-0.5, 0.5, -1.50) Ni	(1.5, -1.5, -2.0) Fe	(-1.0, 0.0, 1.0) Fe	(0.5, -2.0, 0.5) Co
	(-1.0, 1.0, -1.0) Cr	(0.5, -1.0, -0.5) Co	(-1.5, 0.5, 1.5) Cr	(1.0, -3.0, 0.0) Co

(0.0, 0.0, -1.0) Fe	(0.5, -0.5, -0.0) Cr	(-2.0, 1.0, 2.0) Ta	(1.0, -2.5, 0.5) Ta
(-0.5, 0.5, -0.5) Fe	(1.0, -1.0, -0.0) Ni	(-0.5, -0.5, 1.5) Ta	(1.0, -2.0, 1.0) Cr
(1.0, 2.0, -1.0) Fe	(0.5, -1.0, -1.5) Ta	(-1.0, 0.0, 2.0) Ni	(1.5, -3.0, 0.5) Cr
(0.0,2.0, -2.0) Co	(0.5, -0.5, -1.0) Co	(-1.5, 0.5, 2.5) Co	(1.5, -1.5, 1.0) Co
(0.5, 0.5, -2.5) Ni	(0.0, -1.5, -0.5) Ta	(0.0, -1.0, 2.0) Co	(2.0, -2.5, 0.5) Fe
(0.0, 1.0, -2.0) Co	(1.0, -1.0, -1.0) Cr	(-0.5, -0.5, 2.5) Fe	(0.5, -1.5, 0.0) Cr
(-0.5, 1.5, -1.5) Ta	(0.5, -0.5, -2.0) Ni	(-1.0, 0.0, 3.0) Cr	(0.5, -1.0, 0.5) Ni
(0.5, 0.5, -1.5) Ni	(0.0, -1.5, -1.5) Ni	(-1.5, 0.5, 3.5) Ni	(1.0, -2.0, 0.0) Fe
(-0.0, 1.0, -1.0) Fe	(1.0, -1.0, -2.0) Co	(-0.5, 0.5, 0.5) Fe	(1.0, -1.5, 0.5) Ni
(-0.5, 1.5, -0.5) Cr	(1.0, -0.5, -1.5) Ta	(-1.0, 1.0, 1.0) Ni	(1.5, -2.5, 0.0) Fe
(0.5, 0.5, -0.5) Co	(0.5, -1.5, -1.0) Fe	(-1.5, 1.5, 1.5) Ni	(1.5, -2.0, 0.5) Ni
(0.0,1.0, 0.0) Ni	(1.5, -1.0, -1.5) Co	(0.0, 0.0, 1.0) Fe	(2.0, -3.5, 0.5) Cr
(-0.5, 1.5, 0.5) Cr	(1.0, -0.5, -1.5) Ta	(-0.5, 0.5, 1.5) Ta	(2.0, -1.5, 0.5) Ta
(0.5, 1.5, -1.5) Ta	(0.5, -1.5, -1.0) Fe	(-1.0, 1.0, 2.0) Co	(0.5, -0.5, 0.0) Fe
(0.0, 2.0, -1.0) Ni	(1.5, -0.5, -1.0) Co	(0.5, -0.5, 1.5 Co	(1.0, -1.5, 0.0) Co
(1.0, 1.0, -1.0) Fe	(1.0, -1.5, -0.5) Fe	(0.0, 0.0, 2.0) Ta	(1.0, -1.0, 0.0) Fe
(0.5, 1.5, -0.5) Ta	(0.5, -1.5, -2.0) Cr	(-0.5, 0.5, 2.5) Cr	(1.0, -0.5, 0.5) Ni
(0.0, 2.0, 0.0) Cr	(1.5, -0.5, -2.0) Ni	(-0.5, 1.5, 1.5) Cr	(1.5, -1.5, 0.0) Ni
(0.5, 2.5, -0.5) Cr	(1.0, -1.5, -1.5) Cr	(0.5, 0.5, 1.5) Co	(1.5, -1.0, 0.5) Ta
		(0.0, 1.0, 2.0) Fe	(2.0, -2.0, 0.0) Ta

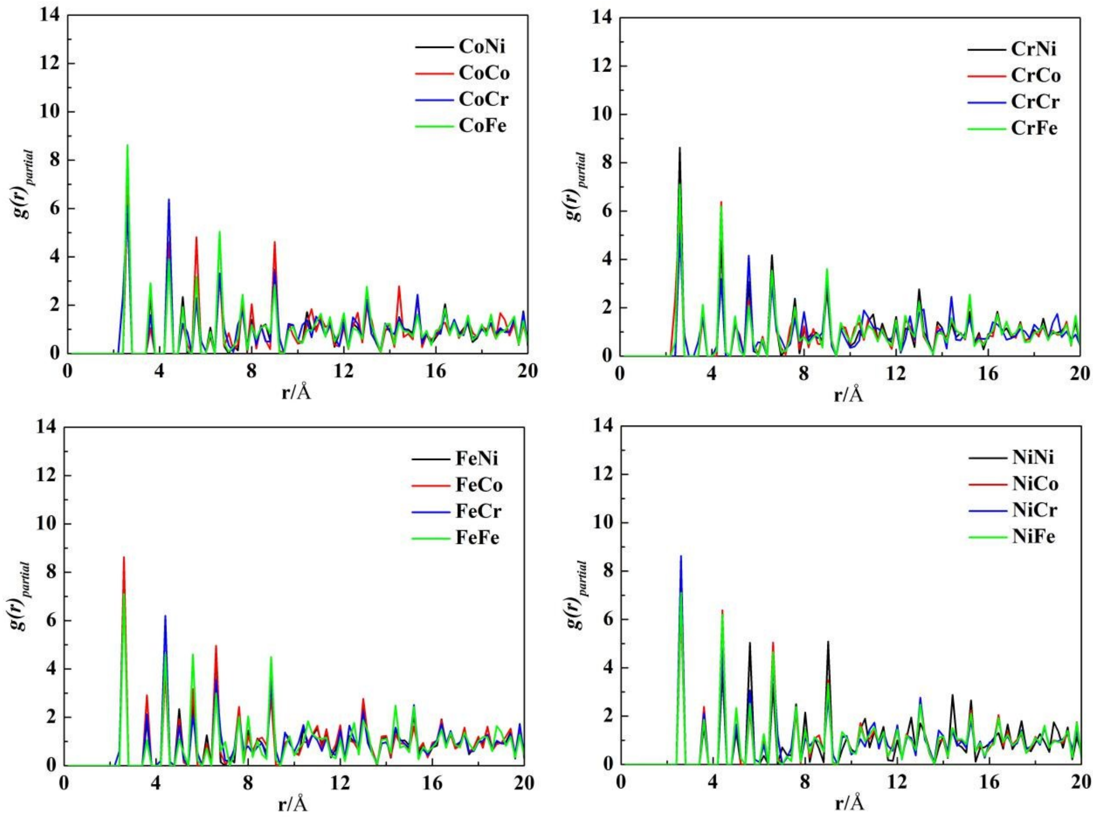

Fig. S1 Partial pair distribution function of *fcc* CoCrFeNi alloy.

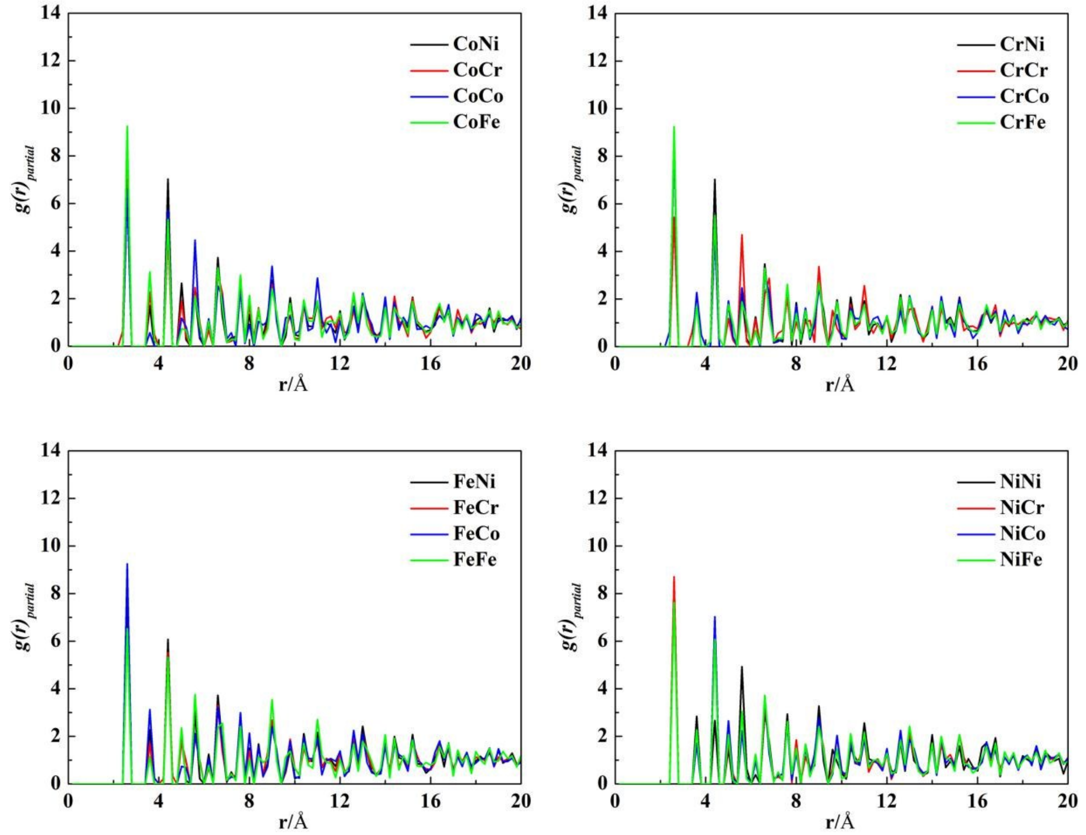

Fig. S2 Partial pair distribution function of *bcc* CoCrFeNi alloy.

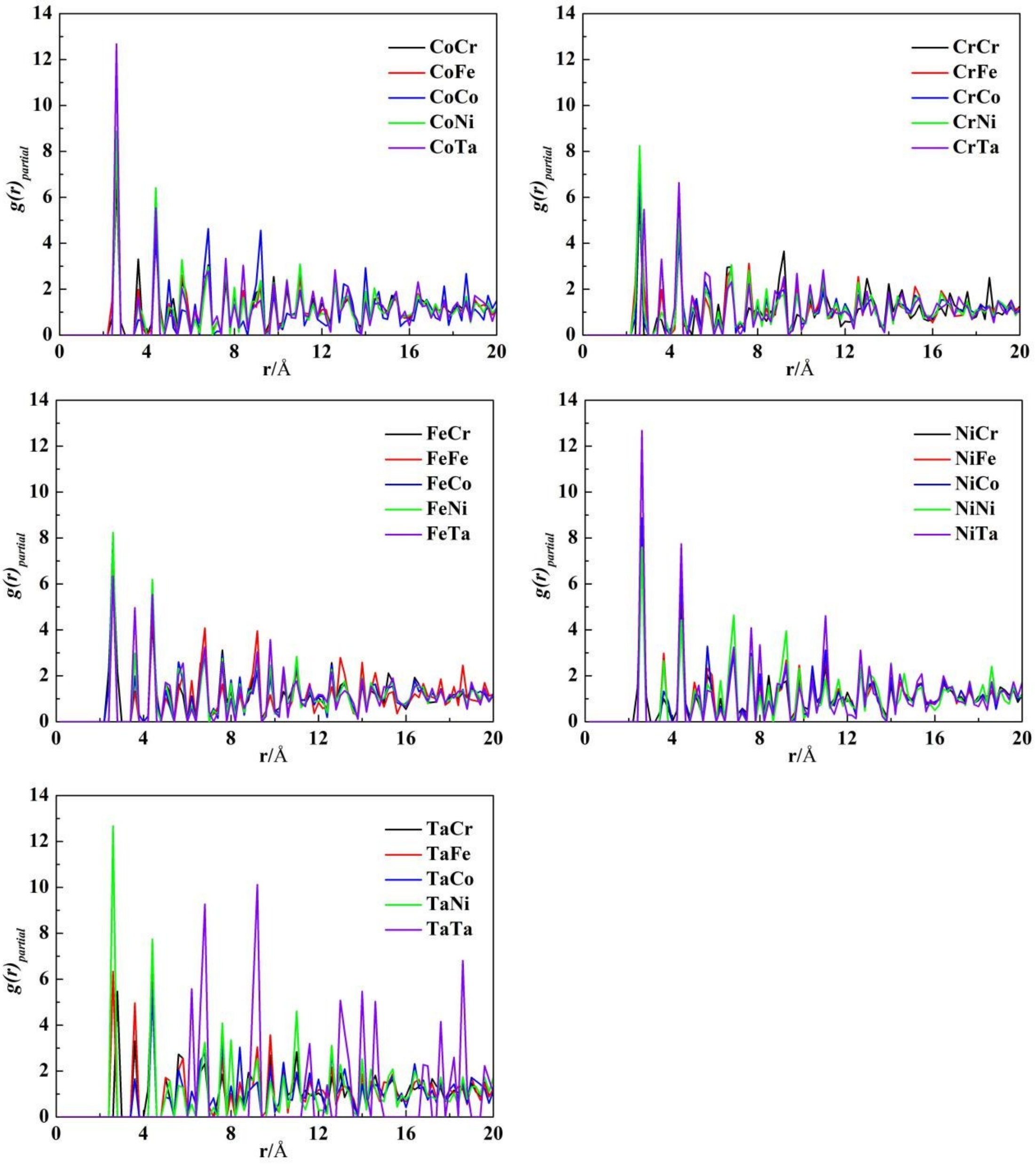

Fig. S3 Partial pair distribution function of *fcc* $CoCrFeNiTa_{0.2}$ alloy.

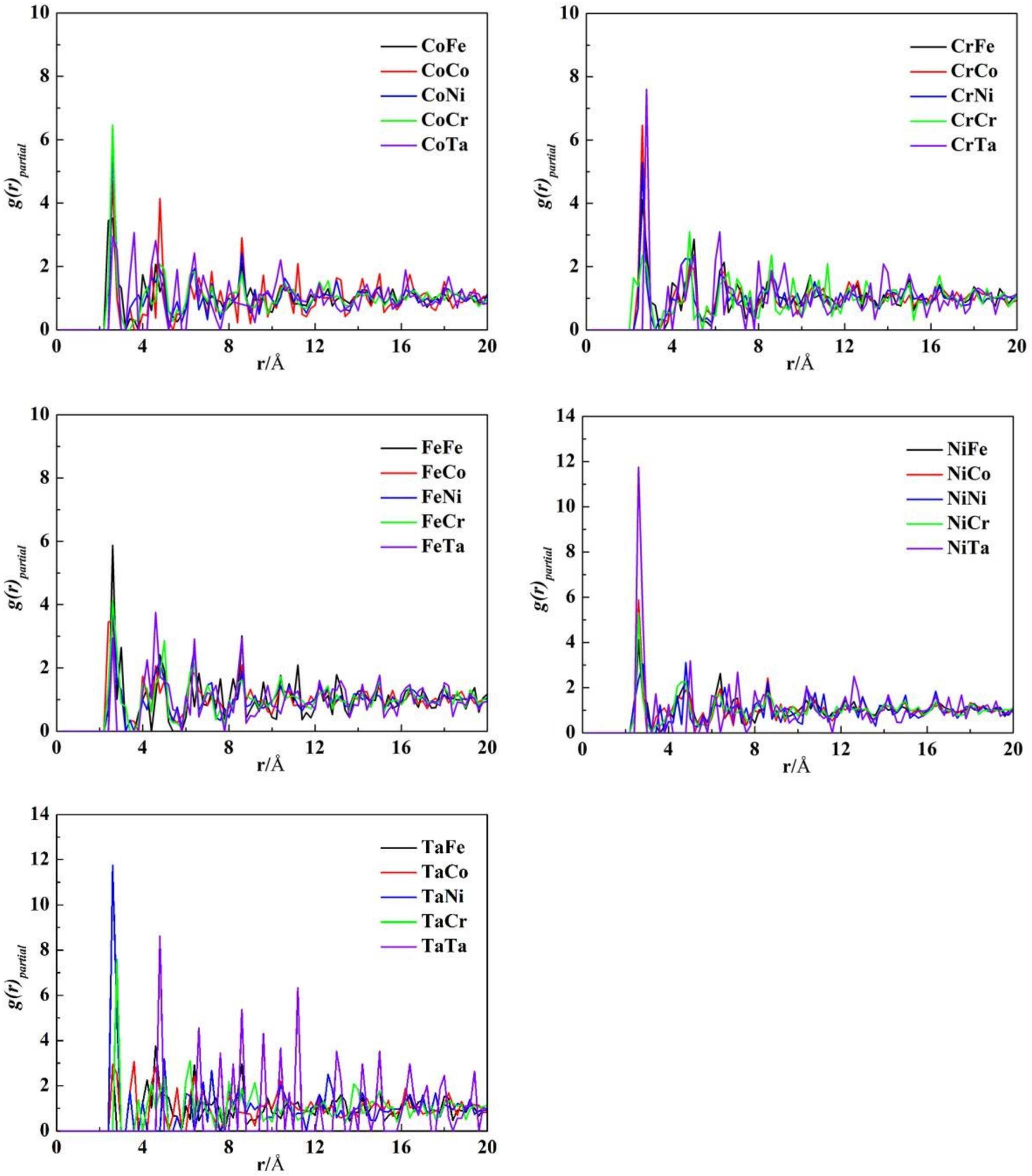

Fig. S4 Partial pair distribution function of *bcc* $CoCrFeNiTa_{0.2}$ alloy.

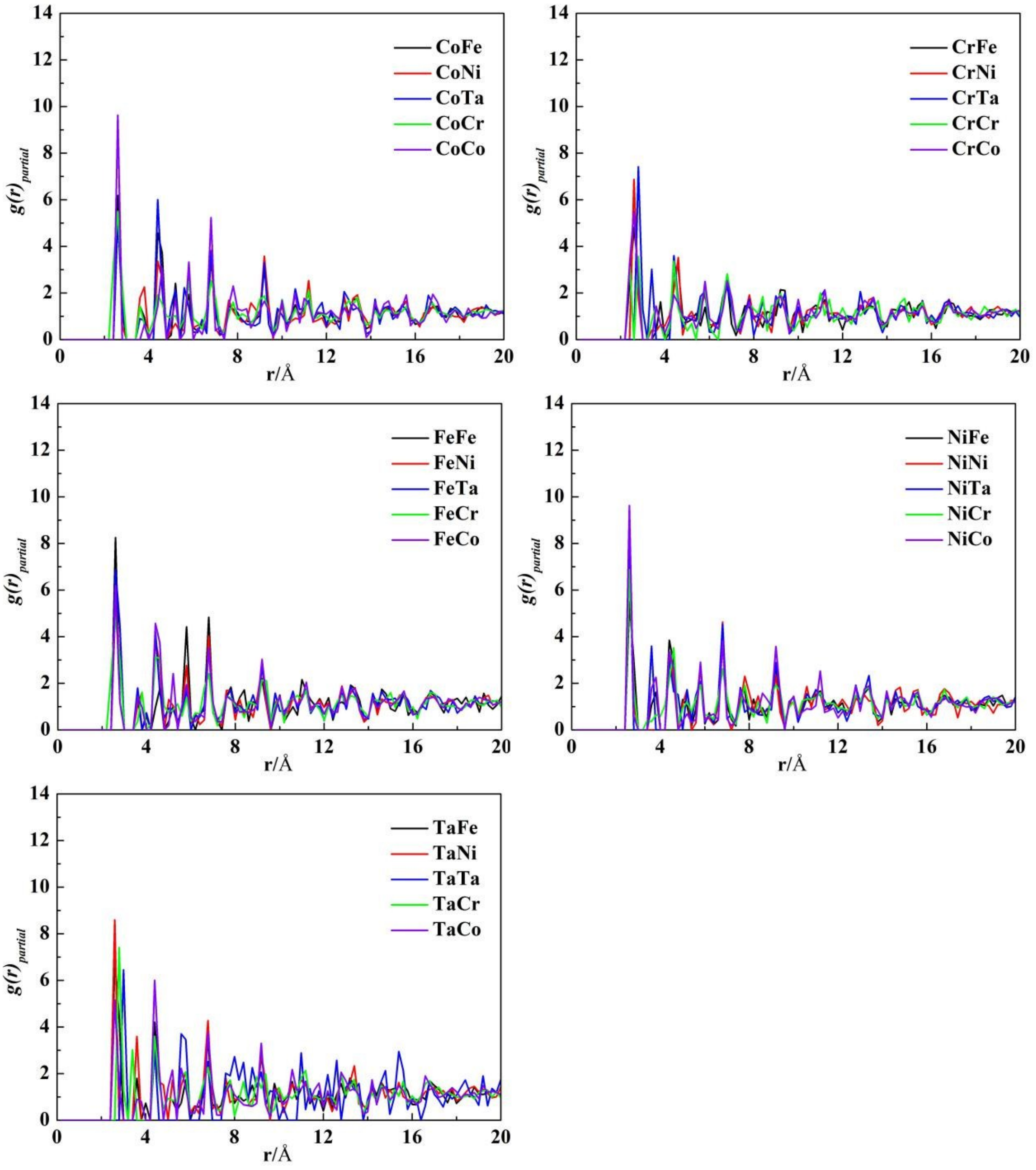

Fig. S5 Partial pair distribution function of *fcc* $CoCrFeNiTa_{0.4}$ alloy.

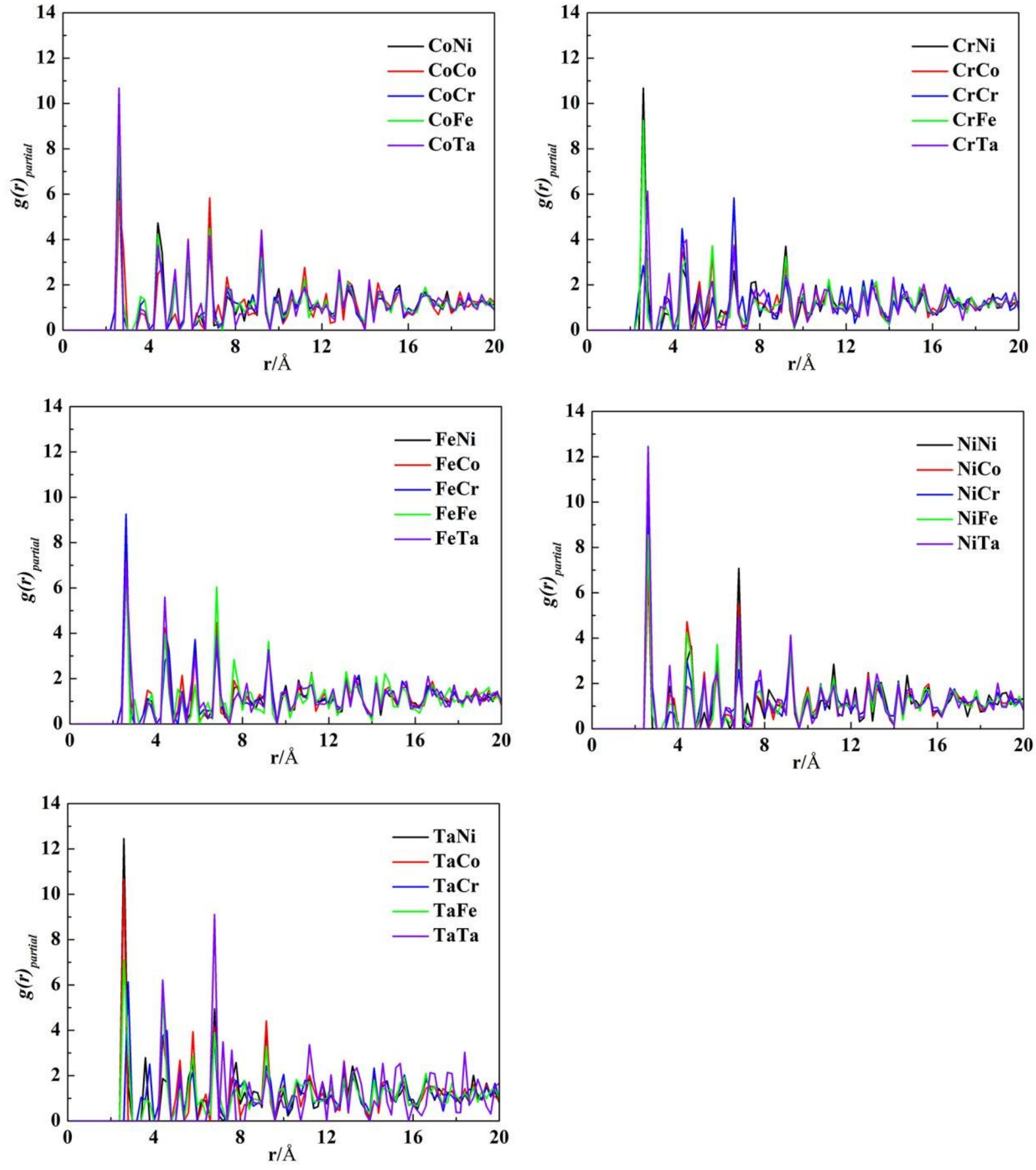

Fig. S6 Partial pair distribution function of *bcc* $CoCrFeNiTa_{0.4}$ alloy.

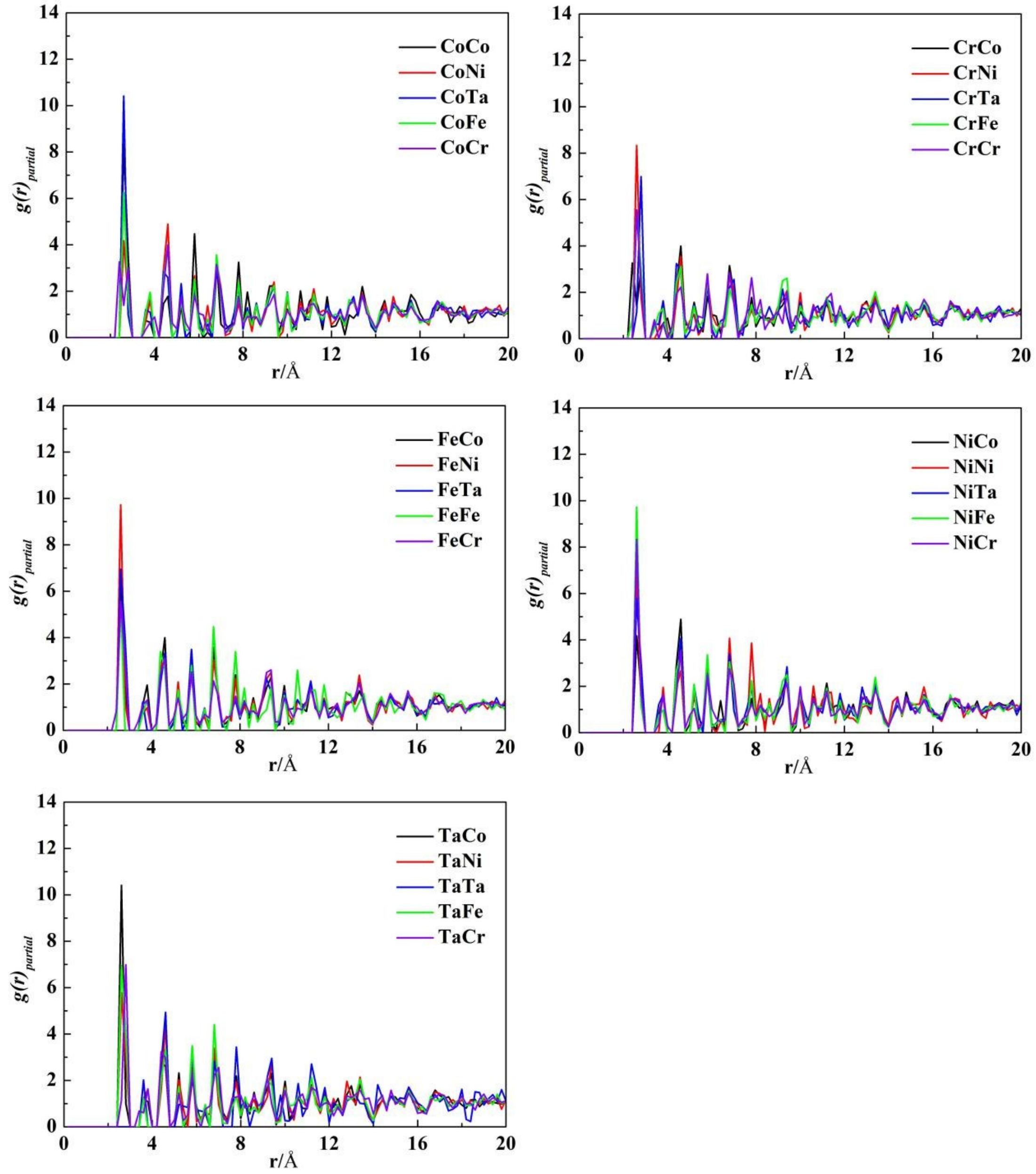

Fig. S7 Partial pair distribution function of *fcc* $CoCrFeNiTa_{0.6}$ alloy.

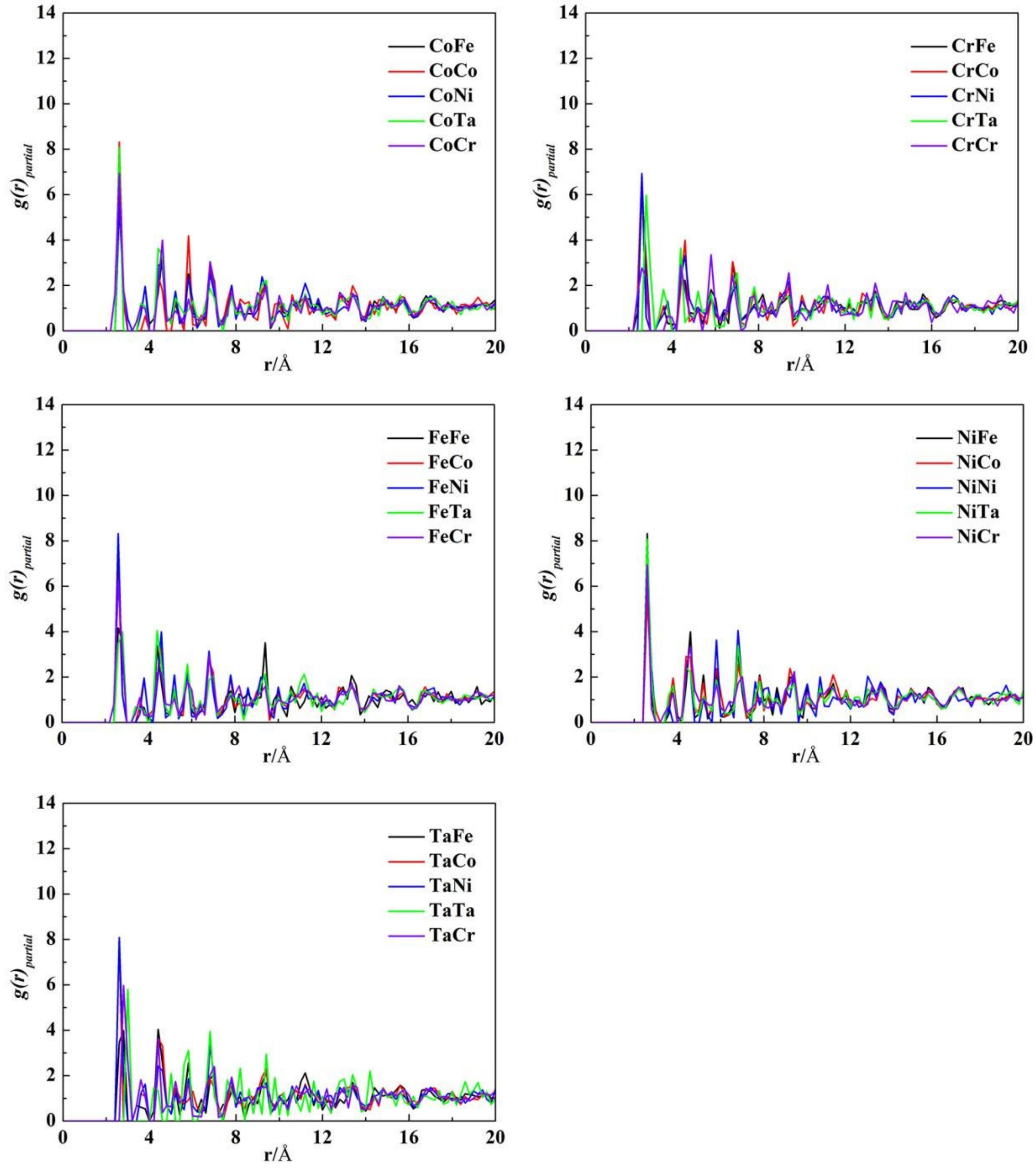

Fig. S8 Partial pair distribution function of *bcc* $CoCrFeNiTa_{0.6}$ alloy.

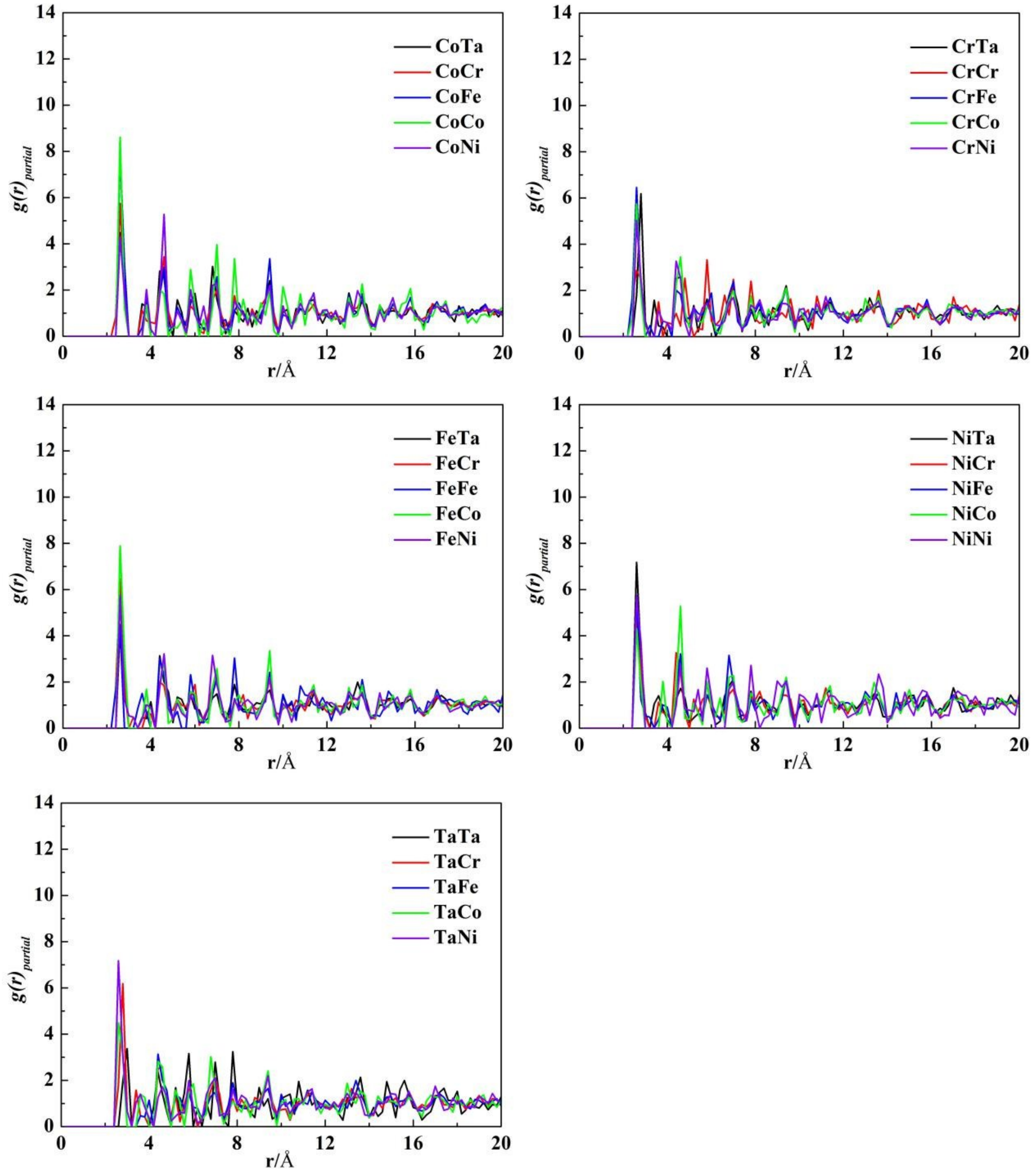

Fig. S9 Partial pair distribution function of *fcc* $CoCrFeNiTa_{0.8}$ alloy.

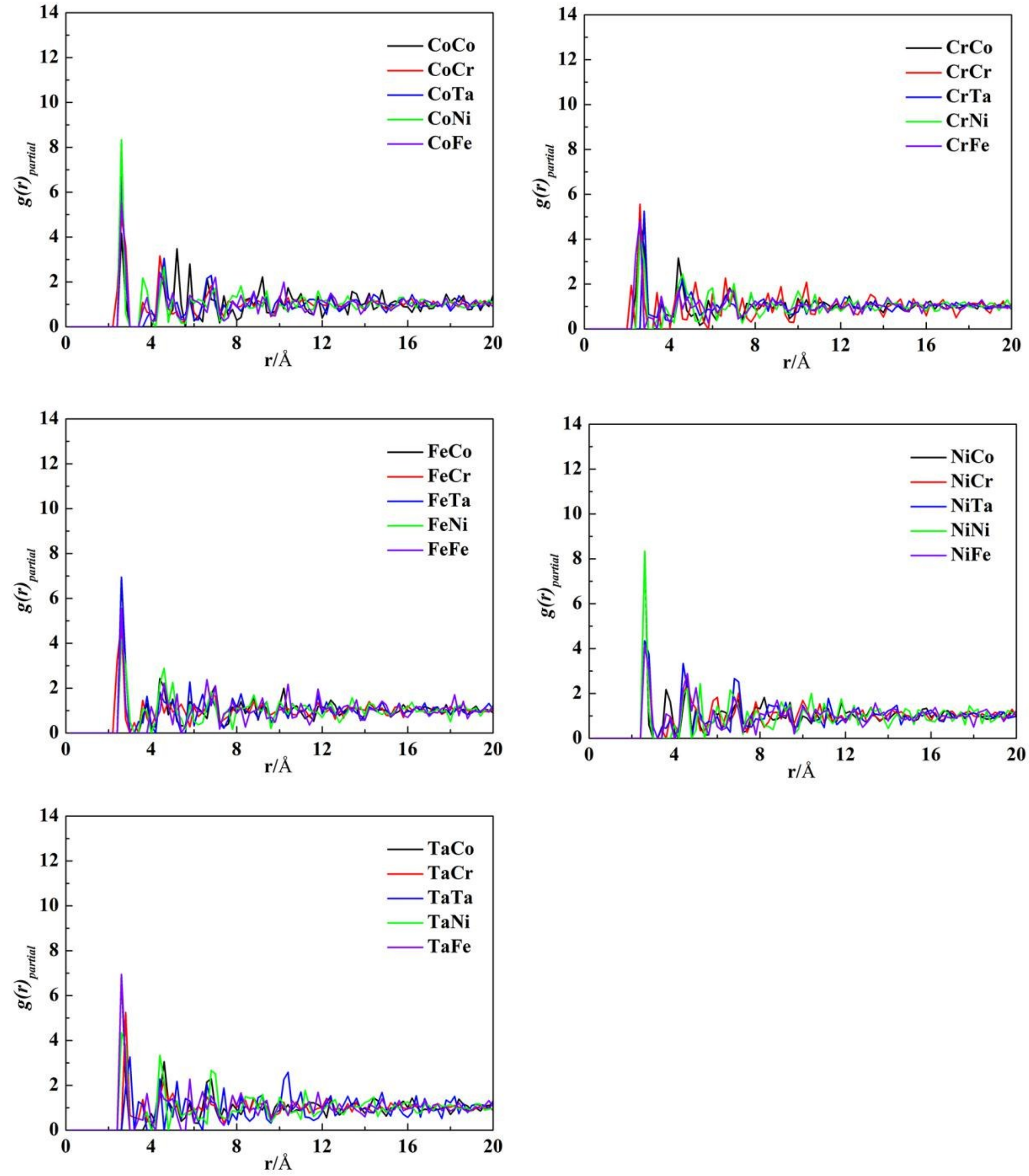

Fig. S10 Partial pair distribution function of *bcc* $CoCrFeNiTa_{0.8}$ alloy.

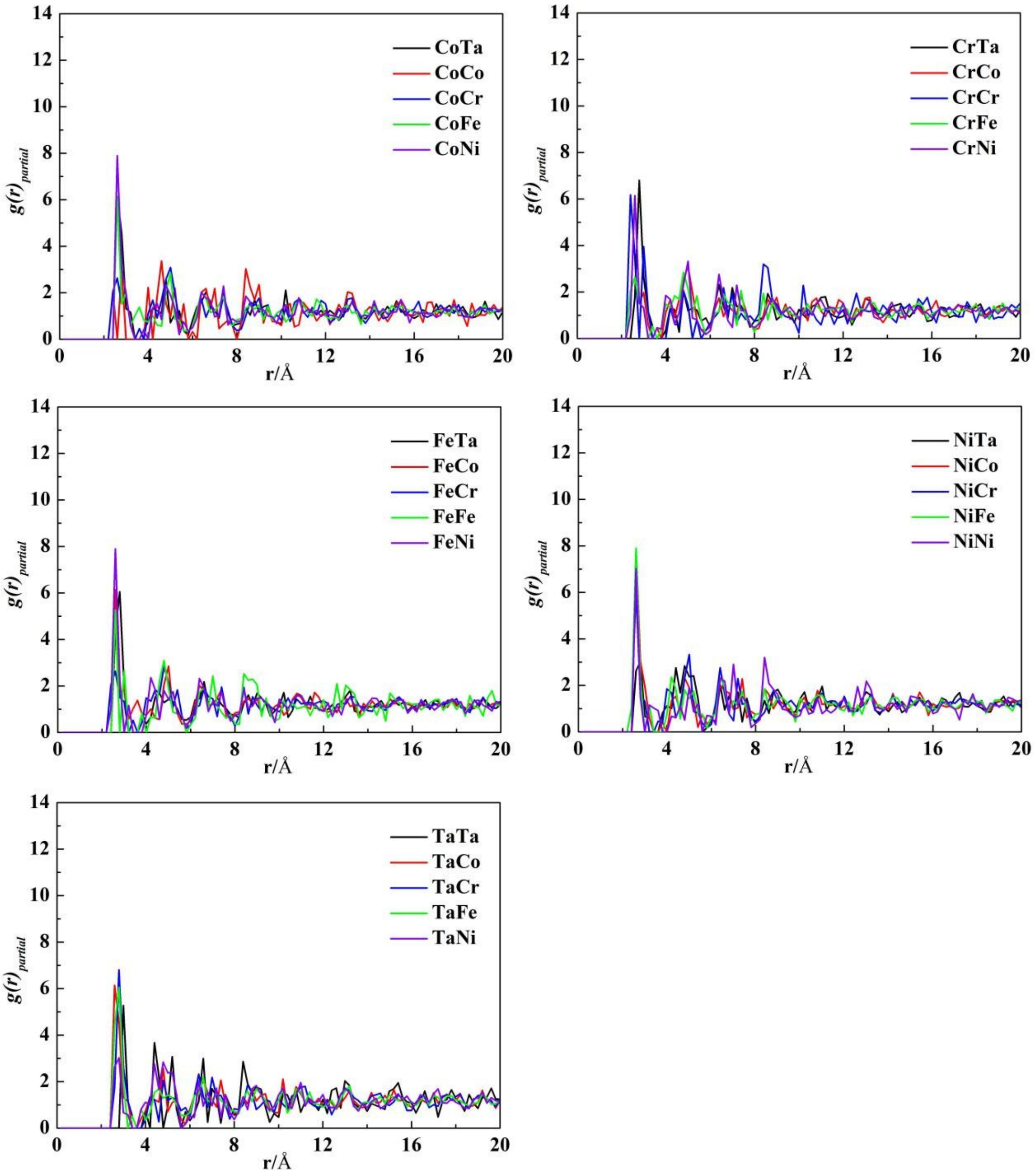

Fig. S11 Partial pair distribution function of *fcc* $CoCrFeNiTa_{1.0}$ alloy.

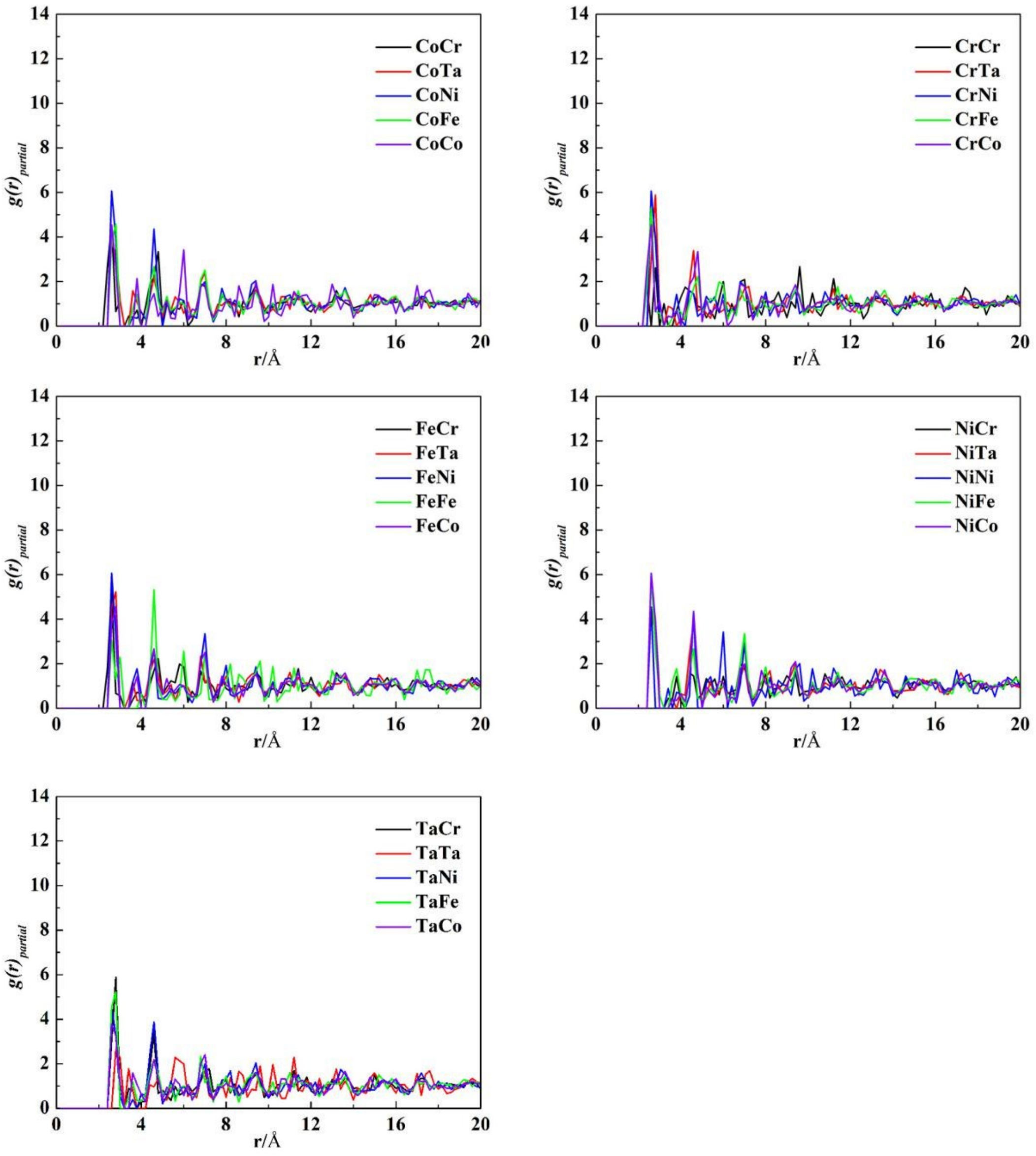

Fig. S12 Partial pair distribution function of *bcc* $CoCrFeNiTa_{1.0}$ alloy.

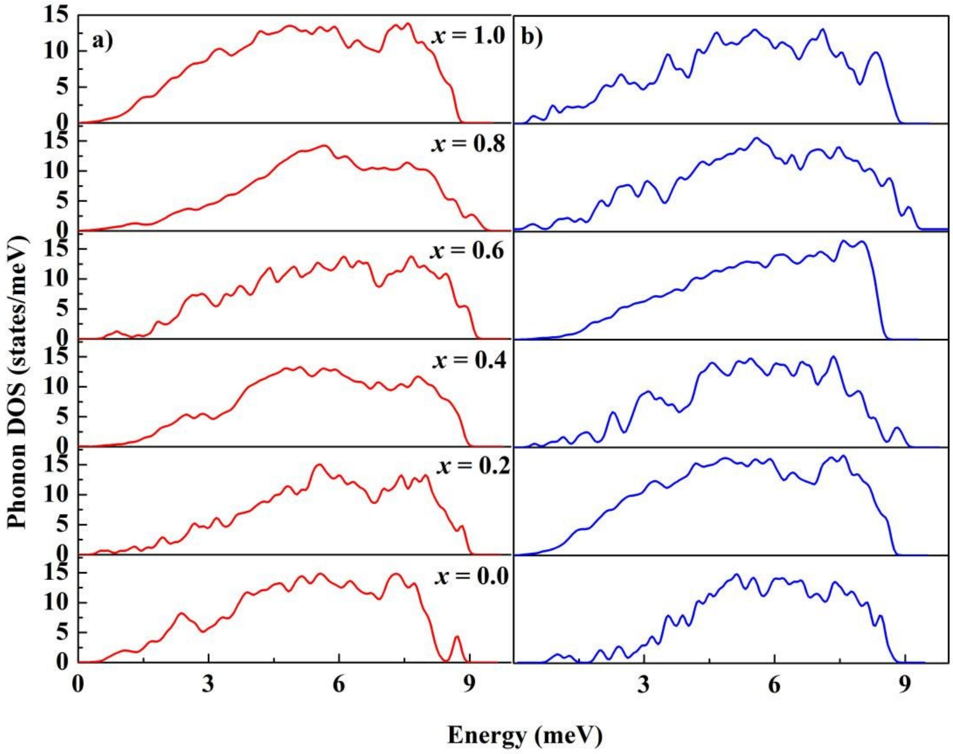

Fig. S13 Phonon density of states for *fcc* (left) and *bcc* (right) structures. *x* represents the Ta content in $CoCrFeNiTa_x$ alloys.

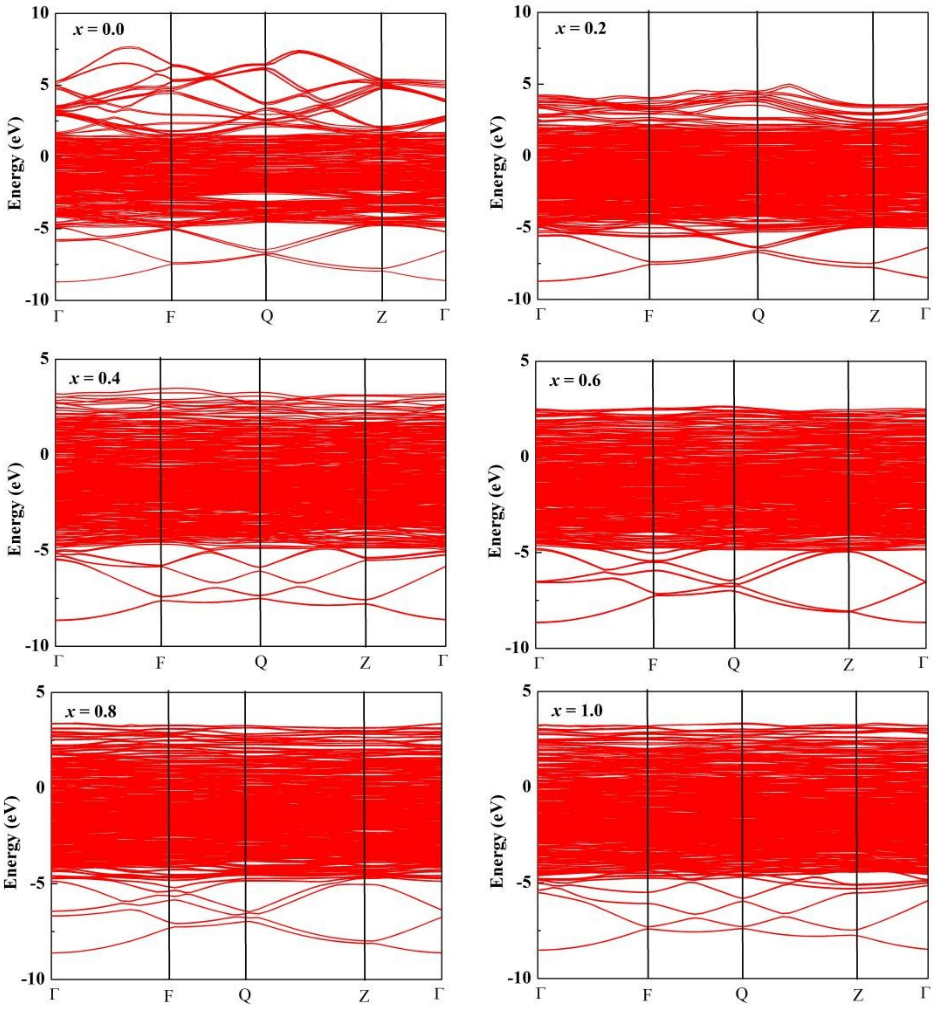

Fig. S14 Band structures of *fcc* $CoCrFeNiTa_x$

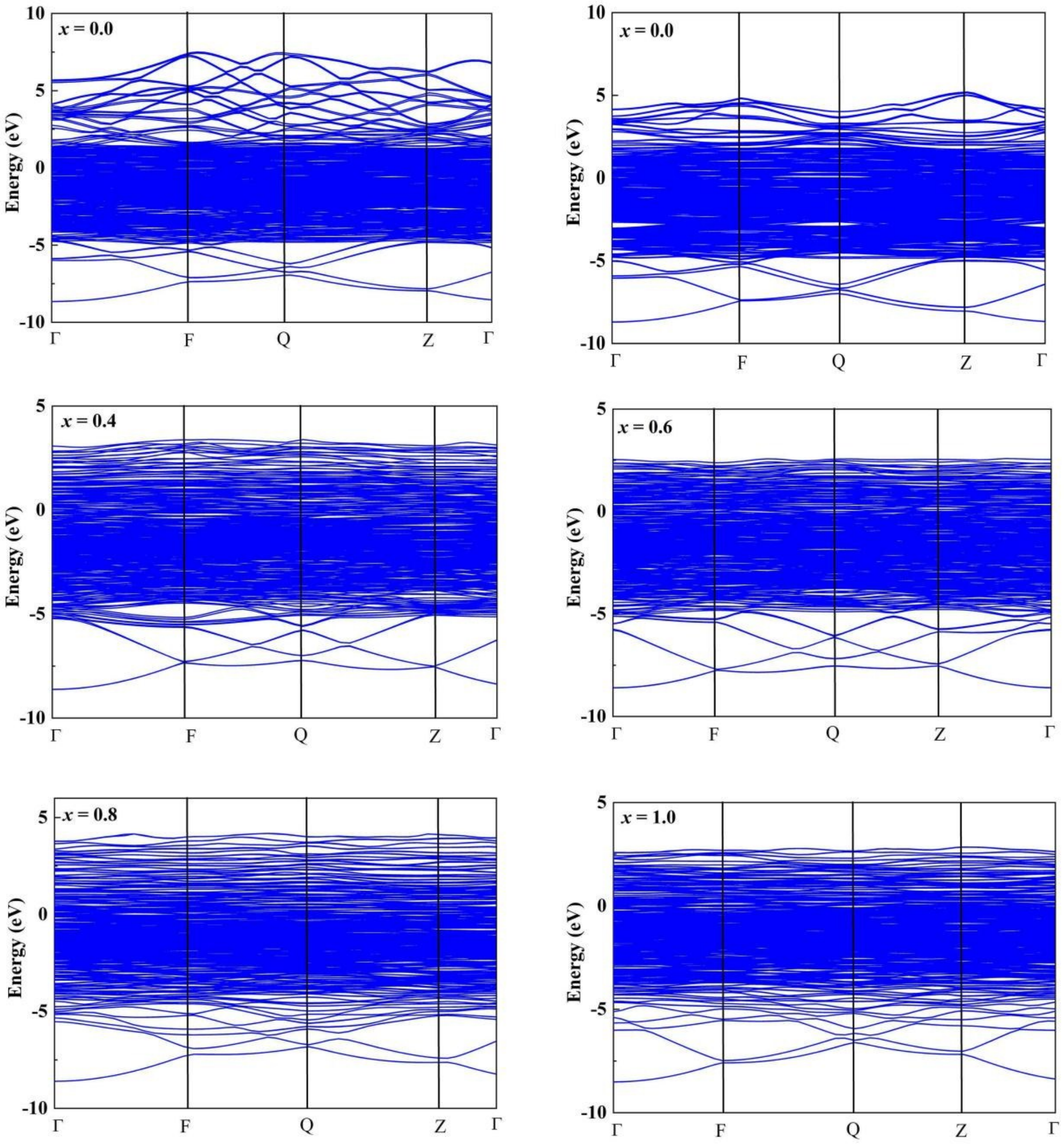

Fig. S15 Band structures of *bcc* $CoCrFeNiTa_x$

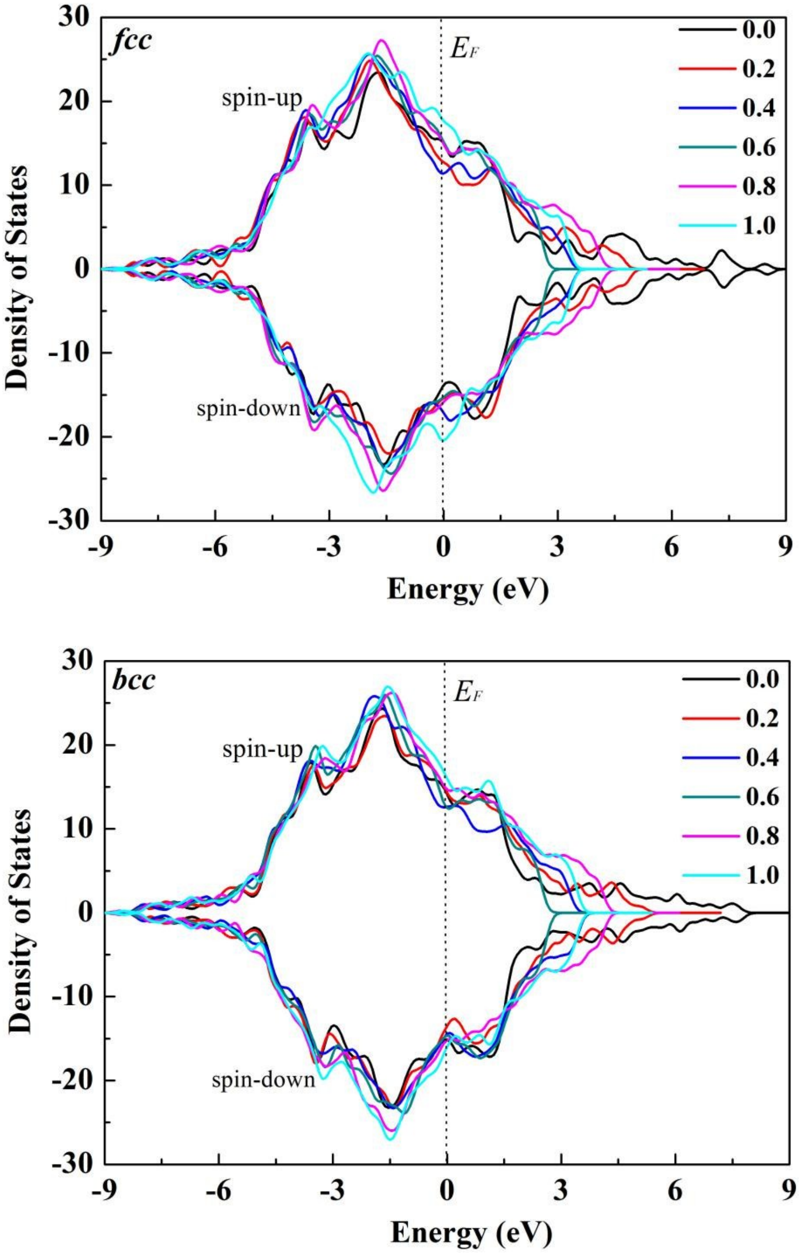

Fig. S16 Density of states of *fcc* and *bcc* $CoCrFeNiTa_x$

Appendix:

According to Boltzmann's hypothesis,[1,2] the configurational entropy of an n-element regular solution is as follows:

$$\Delta S_{con} = - R\sum_{i=1}^{n}(C_i lnC_i) \qquad (1)$$

where C_i is mole percent of element *i*, $\sum_{i=1}^{n} C_i = 1$, and R is the ideal gas constant.

1 R.A. Swalin, *Wiley*, New York, 1972, 35-41.

2 X. Yang and Y. Zhang, *Mater. Chem. Phys.*, 2012, 132, 233-238.
